# Gene therapy ameliorates spontaneous seizures associated with cortical neuron loss in a *Cln2^R207X^* mouse model

**DOI:** 10.1172/JCI165908

**Published:** 2023-06-15

**Authors:** Keigo Takahashi, Elizabeth M. Eultgen, Sophie H. Wang, Nicholas R. Rensing, Hemanth R. Nelvagal, Joshua T. Dearborn, Olivier Danos, Nicholas Buss, Mark S. Sands, Michael Wong, Jonathan D. Cooper

**Affiliations:** 1 Department of Pediatrics,; 2 Department of Neurology, and; 3 Department of Medicine, Washington University School of Medicine, St. Louis, Missouri, USA.; 4 REGENXBIO Inc., Rockville, Maryland, USA.; 5 Department of Genetics, Washington University School of Medicine, St. Louis, Missouri, USA.

**Keywords:** Neuroscience, Therapeutics, Lysosomes, Neurodegeneration, Seizures

## Abstract

Although a disease-modifying therapy for classic late infantile neuronal ceroid lipofuscinosis (CLN2 disease) exists, poor understanding of cellular pathophysiology has hampered the development of more effective and persistent therapies. Here, we investigated the nature and progression of neurological and underlying neuropathological changes in *Cln2^R207X^* mice, which carry one of the most common pathogenic mutations in human patients but are yet to be fully characterized. Long-term electroencephalography recordings revealed progressive epileptiform abnormalities, including spontaneous seizures, providing a robust, quantifiable, and clinically relevant phenotype. These seizures were accompanied by the loss of multiple cortical neuron populations, including those stained for interneuron markers. Further histological analysis revealed early localized microglial activation months before neuron loss started in the thalamocortical system and spinal cord, which was accompanied by astrogliosis. This pathology was more pronounced and occurred in the cortex before the thalamus or spinal cord and differed markedly from the staging seen in mouse models of other forms of neuronal ceroid lipofuscinosis. Neonatal administration of adeno-associated virus serotype 9–mediated gene therapy ameliorated the seizure and gait phenotypes and prolonged the life span of *Cln2^R207X^* mice, attenuating most pathological changes. Our findings highlight the importance of clinically relevant outcome measures for judging preclinical efficacy of therapeutic interventions for CLN2 disease.

## Graphical abstract

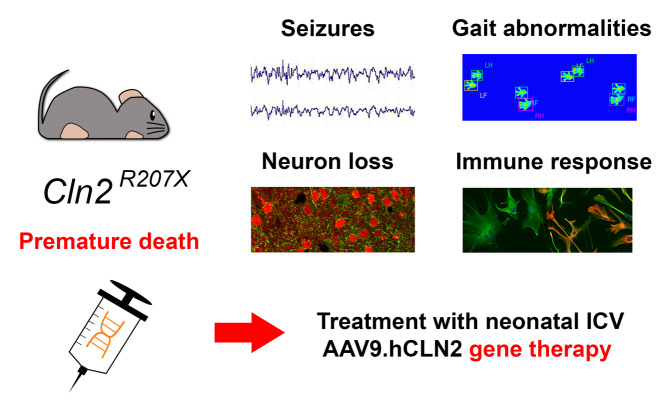

## Introduction

The neuronal ceroid lipofuscinoses (NCLs) are a group of neurodegenerative lysosomal storage disorders affecting children and young adults (1). Classic late infantile NCL, or CLN2 disease, is one of the most common forms of NCL and caused by a deficiency of tripeptidyl peptidase 1 (TPP1) (2–4). Patients with CLN2 disease develop signs typically between 2 and 4 years of age including seizures and ataxia, often accompanied by speech delay (5, 6). These are followed by progressive psychomotor deterioration, and, if untreated, these children die during their teenage years (7). A disease-modifying enzyme replacement therapy (ERT) for CLN2 disease is now available and appears to slow disease progression (8, 9). However, ERT requires invasive biweekly infusions and is not curative. Therefore, a need remains to develop less invasive and more effective therapies for CLN2 disease.

Progress toward this goal has been hampered by a fundamental lack of knowledge of how TPP1 deficiency affects the central nervous system (CNS). Therapy-resistant epilepsy is a major clinical feature in children with CLN2 disease and causes significant morbidity (5–7). Cln2 mice often die suddenly, and the timing appears to be coincident with seizure-like behavior. However, their epileptic seizure phenotype and how this relates to survival have yet to be explored. The histopathological outcome measures used in NCL preclinical studies typically include storage material accumulation, glial activation, and neuron loss (10, 11). The onset and progression of these phenotypes have been characterized in detail in mouse models of multiple other forms of NCL. These studies have revealed temporal and regional differences between the NCLs, information that is important for the targeting of therapies (12–19). Similar neuropathological phenotypes are evident in Cln2-knockout mice at the end stage and have been used to judge efficacy at disease end stage in preclinical studies (20–22). However, the onset, the regional and cellular specificity of these phenotypes, and how they progress over time have yet to be determined.

We have addressed both of these knowledge gaps in a potentially new knockin mouse (*Cln2^R207X^*) that models the most common human disease-causing mutation in the *CLN2* gene (23). We report a robust and potentially novel spontaneous seizure phenotype that is associated with a loss of cortical neurons, including interneurons, and significantly contributes to morbidity. Our data also reveal a range of pronounced differences in the staging of CLN2 disease neuropathology compared with other forms of NCL. Having described these clinically relevant readouts, we then used them as outcome measures to study therapeutic efficacy. We show that a single neonatal intracerebroventricular (ICV) injection of an adeno-associated virus serotype 9 expressing human TPP1 (AAV9.hCLN2) dramatically reduced seizures and interictal epileptiform activity, extended life span, largely preserved gait performance, and partially ameliorated disease-associated neuropathological changes, including interneuron loss, in *Cln2^R207X^* mice.

Taken together, our data represent a thorough characterization of this genetically accurate mouse model of CLN2 disease. These data also provide key quantitative information that is relevant to human disease for judging the efficacy of preclinical interventions. Finally, CNS-directed, AAV9-mediated gene therapy results in a robust therapeutic response based on these outcome measures, including seizures.

## Results

### Electroencephalography monitoring reveals a pronounced seizure phenotype in Cln2^R207X^ mice.

**Figure 1:**
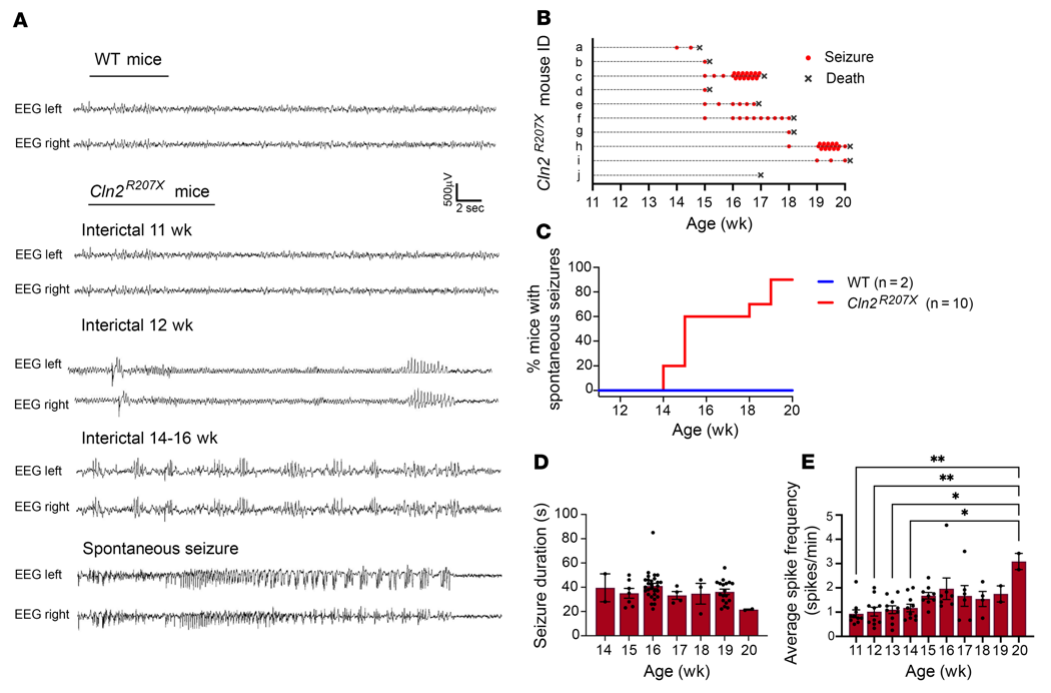
*Cln2^R207X^* mice show a pronounced seizure phenotype. Electroencephalography (EEG) recording reveals epileptiform interictal abnormalities and spontaneous seizures in *Cln2^R207X^* mice. (**A**) Representative EEG traces at different time points in WT and *Cln2^R207X^* mice. (**B**) Time course of spontaneous seizures (red dots) and deaths (black *X*s) in individual *Cln2^R207X^* mice. (**C**) Percentage of mice displaying spontaneous seizures up to 20 weeks in WT (*n* = 2) and *Cln2^R207X^* mice (*n* = 10), with a median age at onset of 15 weeks. (**D**) Average duration of spontaneous seizures in *Cln2^R207X^* mice at each age (in weeks). The overall average seizure duration across all ages was 34.5 seconds. There was no significant difference in seizure duration between *Cln2^R207X^* mice of different ages. (**E**) Average frequency of interictal spikes at each age (in weeks). *P < 0.05, **P < 0.01, 1-way ANOVA with Bonferroni’s correction. Values are shown as mean ± SEM (*n* = 10 *Cln2^R207X^* mice).

Considering the clinical importance of epilepsy in patients with CLN2 disease (5–7), we first performed long-term simultaneous video electroencephalography (EEG) recordings to investigate whether *Cln2^R207X^* mice develop any seizure-related activity and how this may relate to their premature death. WT control mice showed normal EEG background activity consisting of mixed background EEG frequencies and no epileptiform activity (Figure 1A). At 10 weeks of age, *Cln2^R207X^* mice exhibited a similar EEG background compared to WT mice, but at approximately 12 weeks of age, *Cln2^R207X^* mice started to display interictal epileptiform spikes and spike bursts superimposed on a mostly normal background (Figure 1A). A progressive increase in background spiking and clustering of spike bursts occurred over the 12- to 14-week period in *Cln2^R207X^* mice, resulting in generalized EEG slowing, frequent spiking, bursting, and often burst suppression activity by 16 weeks of age (Figure 1A). There was no evident behavioral correlate, such as freezing or myoclonic seizures, with this interictal epileptiform activity. Starting at 14 weeks of age, *Cln2^R207X^* mice developed stereotypical spontaneous electrographic seizures involving a sudden tonic spike discharge, which evolved in frequency and amplitude (Figure 1A). These EEG-recorded seizures were always accompanied by video recorded clinical seizures. These clinical seizures were typically characterized by head bobbing, rearing with forelimb clonus, intermittent tail stiffening and flicking, and occasional generalized convulsive activity that did not change their presentation with disease progression (Supplemental Video 1; supplemental material available online with this article; https://doi.org/10.1172/JCI165908DS1). Further analysis of the temporal relationship between seizure activity and death revealed that 50% of *Cln2^R207X^* mice died after a single seizure or no more than 3 seizures (5/10 mice). A further 20% of mice died after a series of 5–10 individual seizure events spaced over several days (2/10 mice), and 20% of mice died after a closely spaced burst of 15–20 seizure events within 1 week (2/10 mice), with 1 mouse apparently dying in the absence of seizure activity (Figure 1B). Notably, 70% of *Cln2^R207X^* mice died within 3 minutes of their last seizure (Table 1 and Supplemental Video 1). These spontaneous seizures were present in 90% of *Cln2^R207X^* mice, with a median age at onset of 15 weeks (Figure 1C). Spontaneous seizures lasted an average of 34.5 seconds and did not differ significantly in duration with disease progression (Figure 1D). In contrast, the frequency of interictal spikes in *Cln2^R207X^* mice increased over time, being significantly greater at 20 weeks of age compared with younger ages, between 11 and 14 weeks (Figure 1E).

### Late-onset gait abnormalities in Cln2^R207X^ mice.

**Figure 2:**
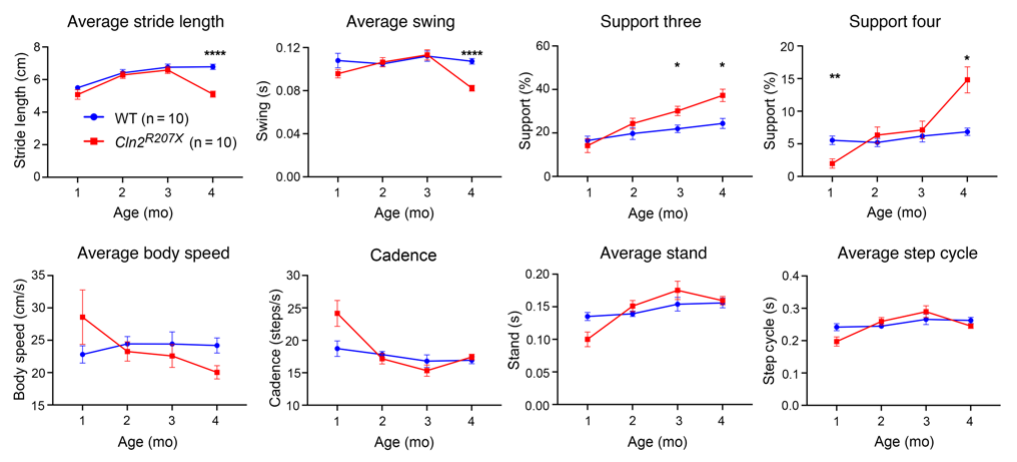
*Cln2^R207X^* mice show late-onset gait abnormalities. CatWalk XT gait analysis reveals significantly shorter average stride length and average swing duration at 4 months and higher proportion of steps supported by 3 or 4 feet at 3–4 months in *Cln2^R207X^* mice (red lines) compared with age-matched WT control mice (blue lines). Other parameters, including average body speed, cadence, average stand time, and average step cycle time, showed no significant change between genotypes. *P < 0.05, **P < 0.01, ****P < 0.0001, 2-way ANOVA with Bonferroni’s correction. Values are shown as mean ± SEM (*n* = 10 mice per group).

Gait disturbances are another major symptom of CLN2 disease and are used to evaluate disease progression in patients (24, 25). Gait abnormalities in *Cln2*-knockout mice have only been previously documented at disease end stage using rudimentary footprint analysis (21). Here, we quantified the nature and progression of gait abnormalities in *Cln2^R207X^* mice using the CatWalk XT apparatus, at monthly intervals alongside age-matched WT controls. Our data revealed that *Cln2^R207X^* mice exhibited significantly shorter average stride length and average swing duration at 4 months and spent more time supporting themselves on 3 and 4 feet from 3–4 months (Figure 2), suggesting a late onset of locomotor deficits. Nevertheless, many other gait parameters, including average body speed, cadence, average stand time, and average step cycle time, were not significantly altered in *Cln2^R207X^* mice (Figure 2).

### Pyramidal neuron loss occurs in the forebrain of Cln2^R207X^ mice before the spinal cord.

**Figure 3:**
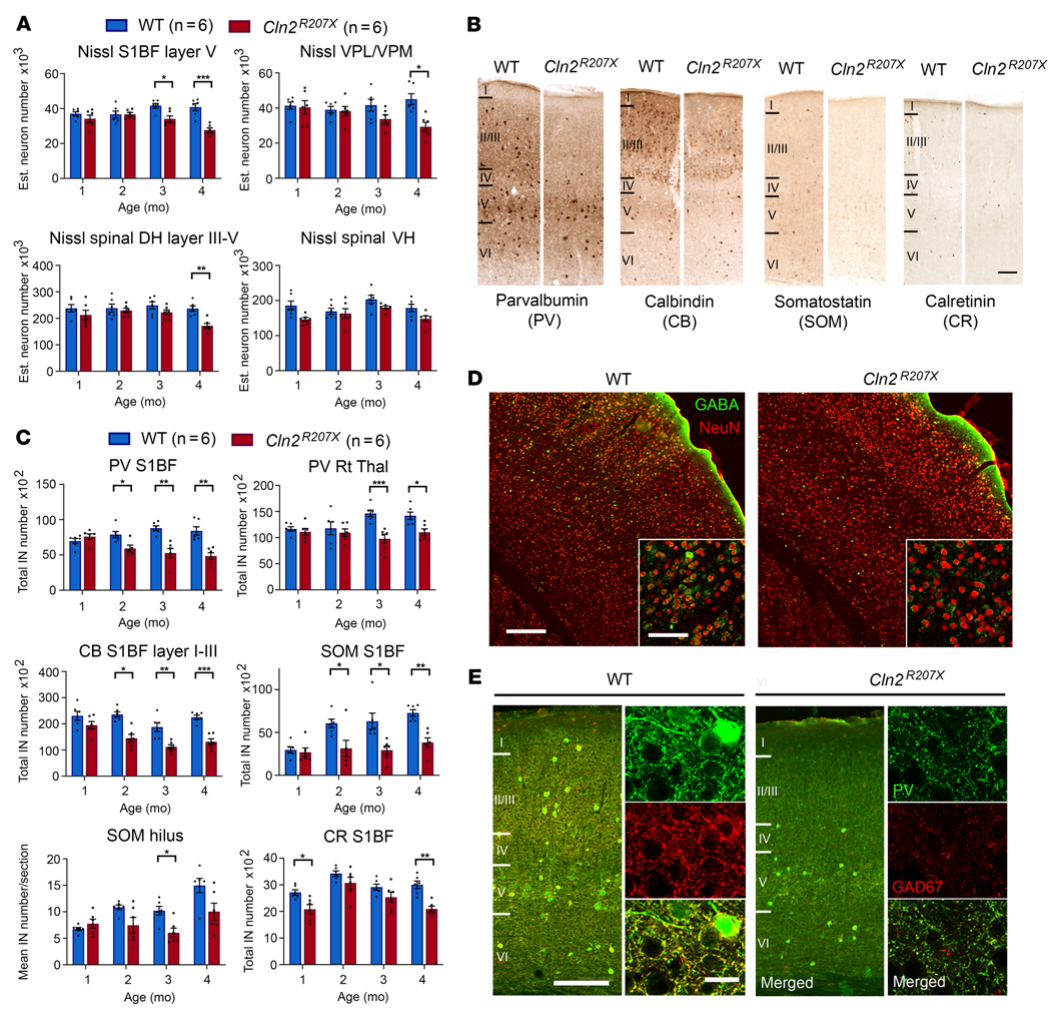
*Cln2^R207X^* mice show cortical Nissl-stained pyramidal neuron loss and marked GABAergic interneuron loss. (**A**) Unbiased stereological counts reveal significant loss of Nissl-stained neurons in layer V of primary somatosensory cortex (S1BF) from 3 months and in ventral posterior thalamic nuclei (VPM/VPL) and lumbar spinal dorsal horn (DH) at 4 months in *Cln2^R207X^* mice (red bars) versus age-matched WT control mice (blue bars) but no significant difference between genotypes in lumbar spinal cord ventral horn (VH). (**B**) Immunoperoxidase staining for parvalbumin (PV), calbindin (CB), somatostatin (SOM), and calretinin (CR) shows fewer interneurons positive for these markers in S1BF of *Cln2^R207X^* mice versus WT mice at 4 months. Scale bar: 200 μm. (**C**) Stereological counts reveal significant loss of PV-, CB-, and SOM-positive S1BF neurons in *Cln2^R207X^* mice (red bars) versus WT mice (blue bars) from 2 months, of PV-positive interneurons in thalamic reticular nucleus (Rt) from 3 months, and of CR-positive interneurons in S1BF at both 1 and 4 months in *Cln2^R207X^* mice. Loss of SOM-positive interneurons in the hippocampal hilus of *Cln2^R207X^* mice was significant only at 3 months. (**D**) Fewer GABA^+^ (green) and NeuN^+^ (red) neurons in S1BF of *Cln2^R207X^* mice at 3 months versus age-matched WT mice. Insets are higher magnification views from layer II/III. Scale bars: 200 μm, 100 μm (insets). (**E**) Decreased immunoreactivity for PV (green) and glutamate decarboxylase 67 (GAD67) (red) terminals and fibers, especially within S1BF layers II/III in *Cln2^R207X^* mice at 3 months, with higher power confocal images from layers II/III (right of each column). Scale bars: 20 μm (right), 200 μm (left). Dots represent values from individual animals. Values are mean ± SEM (*n* = 6 mice per group). *P < 0.05, **P < 0.01, ***P < 0.001, multiple *t* test with Holm-Šídák correction (**A** and **C**).

To explore the etiology of these newly defined seizure phenotypes and gait abnormalities in *Cln2^R207X^* mice, we investigated the onset and progression of neuron loss in the thalamocortical system and spinal cord using unbiased stereological methods. Significant loss of Nissl-stained pyramidal neurons within primary somatosensory cortex (S1BF) layer V was first present in *Cln2^R207X^* mice at 3 months and within ventral posterior nuclei of thalamus (VPM/VPL) only at 4 months of age (Figure 3A). There were also alterations in the morphology of surviving neurons, which often appeared distended with storage material (Supplemental Figure 1A). A significant loss of spinal cord dorsal horn neurons in layer III-V was present only in *Cln2^R207X^* mice at 4 months, with no significant loss of neurons in the ventral horn (layer VI-IX) at any age (Figure 3A). In contrast, no significant changes were observed in cornu ammonis subfield 1 (CA1) hippocampal neurons (Supplemental Figure 2, A and B).

### Loss of GABAergic interneurons and other cortical neurons.

GABA-expressing interneuron dysfunction and loss have been linked to epileptogenesis (26, 27). To determine the relationship between altered EEG activity and potential interneuron loss, we stained *Cln2^R207X^* forebrain sections for calcium-binding proteins or neuropeptides expressed predominantly by interneuron populations, including parvalbumin (PV), calretinin (CR), and somatostatin (SOM). We also stained for calbindin (CB), which is expressed principally by interneurons but also by some excitatory cortical neurons (28). There was a progressive decline in the number of PV-, CB-, and SOM-positive interneurons in *Cln2^R207X^* mice as early as 2 months in S1BF and PV-positive interneurons from 3 months in the thalamic reticular nucleus (Rt), which receives excitatory inputs from both corticothalamic and thalamocortical neurons (29) (Figure 3, B and C). Immunostaining for GABA revealed fewer GABA^+^NeuN^+^ neurons in S1BF of *Cln2^R207X^* mice at 3 months compared with age-matched WT mice (Figure 3D). The distribution of PV-positive fibers and terminals within S1BF of *Cln2^R207X^* mice was drastically diminished within layer II/III of S1BF as early as 3 months (Figure 3E). This layer-specific effect on PV terminals within S1BF was precisely mirrored in the same sections simultaneously stained for GAD67, an enzyme that metabolizes glutamate into GABA (Figure 3E). To investigate the percentage of cortical CB-positive neurons that are GABAergic and to assess whether this is affected by disease, we also co-immunostained brain sections from 4-month-old mice for GABA and CB. Our analysis revealed that the expression of GABA in CB-positive neurons within S1BF was layer specific, varying from approximately 1% in layer IV to 40% in layers V and VI, which did not significantly differ between WT and *Cln2^R207X^* mice (Supplemental Figure 1C), suggesting that CB-positive excitatory neurons are also affected in *Cln2^R207X^* mice. The number of CR-positive interneurons was also significantly reduced in *Cln2^R207X^* mice at 1 and 4 months (Figure 3B), suggesting both developmental and degenerative changes in CR-positive interneurons. In contrast, only minimal changes were observed in the number of any interneuron population within the hippocampus of *Cln2^R207X^* mice (Figure 3C and Supplemental Figure 2, A and B).

To investigate potential effects upon neurotransmitter levels, we quantified concentrations of glutamate, glutamine, and GABA using liquid chromatography–tandem mass spectrometry (LC-MS/MS) in *Cln2^R207X^* cortices at 3 months, immediately before the onset of spontaneous seizures (Figure 1C). There were no significant changes in the cortical concentrations of these neurotransmitters between genotypes (Supplemental Figure 1B).

### Early and widespread storage material accumulation in the CNS of Cln2^R207X^ mice.

**Figure 4:**
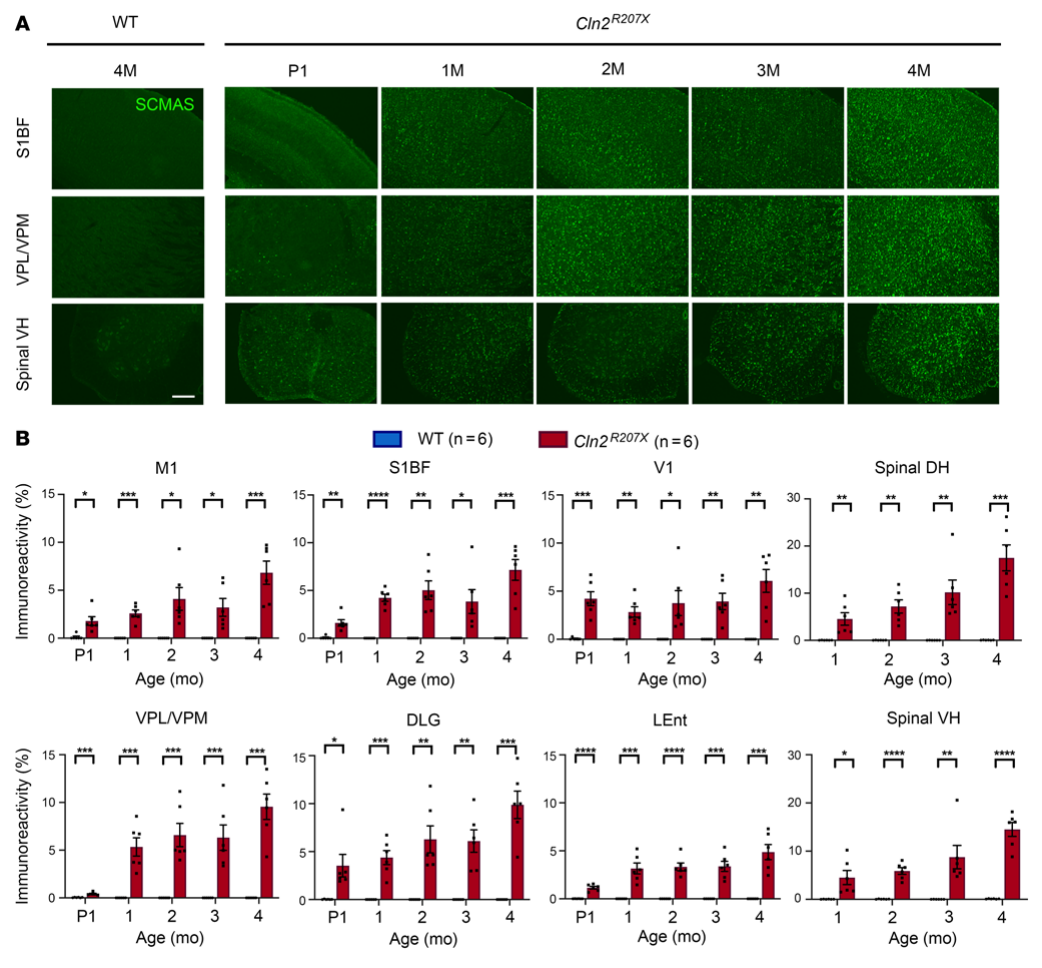
*Cln2^R207X^* mice show early widespread lysosomal storage material accumulation across the CNS. (**A**) Immunostaining for subunit c of mitochondrial ATP synthase (SCMAS, green) reveals the widespread increase in SCMAS accumulation in the primary somatosensory cortex (S1BF), ventral posterior nuclei of thalamus (VPM/VPL), and lumbar spinal ventral horn (VH) in *Cln2^R207X^* mice compared with WT control mice as early as neonatal P1. Scale bar: 200 μm. (**B**) Quantitative analysis of SCMAS immunoreactivity via thresholding image analysis in the same brain regions verifies the early onset of SCMAS accumulation across many CNS regions in *Cln2^R207X^* mice (red bars) compared with age-matched WT control mice (blue bars). Dots represent values from individual animals. *P < 0.05, **P < 0.01, ***P < 0.001, ****P < 0.0001, multiple *t* test with Holm-Šídák correction. Values are shown as mean ± SEM (*n* = 6 mice per group).

Storage material accumulation is a characteristic CNS neuropathological hallmark of the NCLs (10, 11). Thresholding analysis of immunostaining for subunit c of mitochondrial ATPase (SCMAS), a major protein component of this storage material in CLN2 disease (30), revealed a significant increase in SCMAS immunoreactivity at 1 month in all regions of the *Cln2^R207X^* CNS (Figure 4, A and B). Analyzing neonatal *Cln2^R207X^* brains and spinal cords revealed that a pronounced and significant accumulation of SCMAS within most CNS regions was already present at postnatal day 1 (P1) (Figure 4, A and B).

### Localized early microglial activation precedes astrogliosis in the Cln2^R207X^ mouse.

**Figure 5:**
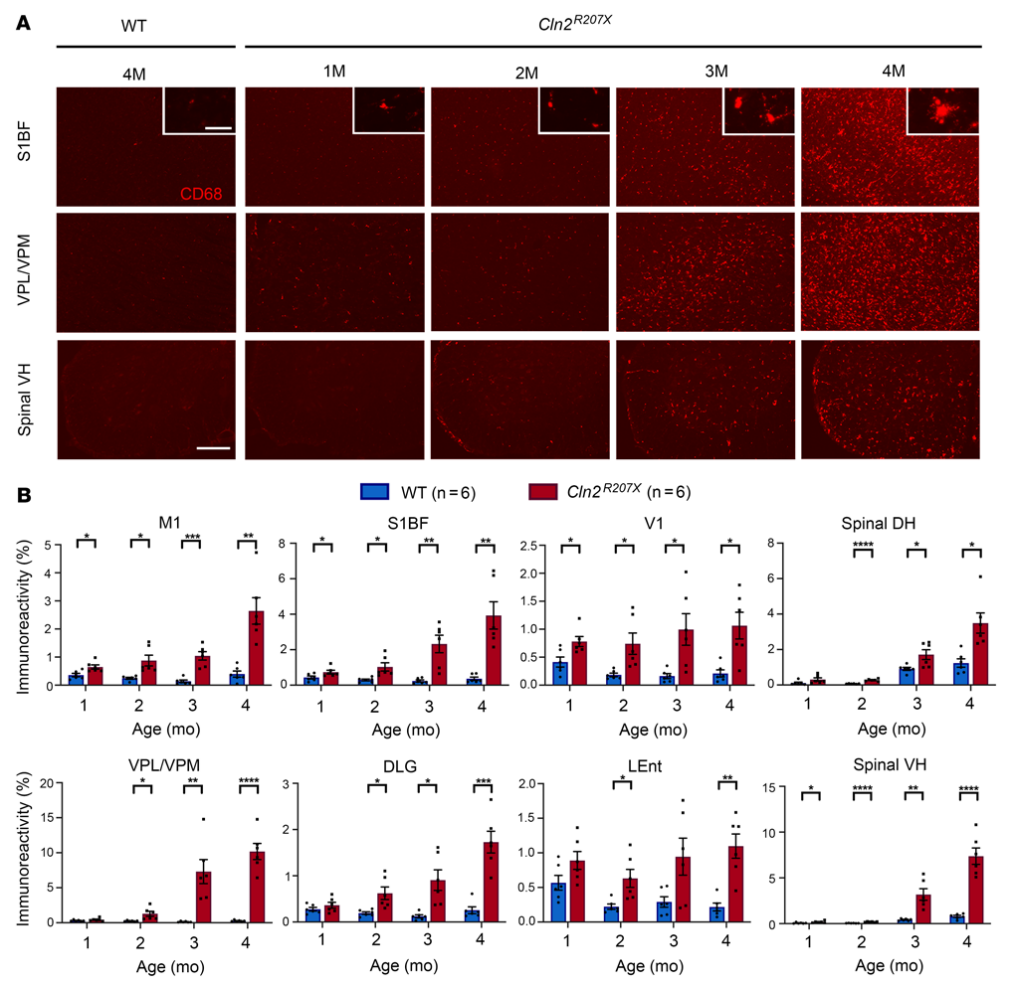
*Cln2^R207X^* mice show localized early microglial activation. (**A**) Immunostaining for the microglial marker cluster of differentiation (CD68, red) reveals the pronounced increase in abundance and staining intensity of CD68 immunoreactivity with disease progression in the primary somatosensory cortex (S1BF), ventral posterior nuclei of thalamus (VPM/VPL), and lumbar spinal ventral horn (VH) in *Cln2^R207X^* mice compared with WT control mice. Corresponding changes in microglial morphology are revealed in higher power insets. Scale bars: 200 μm, 20 μm (*insets*). (**B**) Quantitative thresholding image analysis of CD68 immunoreactivity verifies the progressive nature of microglial activation in *Cln2^R207X^* mice (red bars) beginning in the primary motor cortex (M1), S1BF, primary visual cortex (V1), and spinal VH at 1 month of age, followed by microglial activation in the VPM/VPL, dorsolateral geniculate nucleus (DLG), lateral entorhinal cortex (LEnt), and lumbar spinal dorsal horn (DH) starting at 2 months compared with age-matched WT control mice (blue bars). Dots represent values from individual animals. *P < 0.05, **P < 0.01, ***P < 0.001, ****P < 0.0001, multiple *t* test with Holm-Šídák correction. Values are shown as mean ± SEM (*n* = 6 mice per group).

Microglial activation and astrogliosis are both key neuropathological hallmarks of the NCLs and are especially pronounced within the thalamocortical system (10, 31) but have been documented only at disease end stage in *Cln2^R207X^* mouse brains (23). Therefore, we immunostained brain and spinal sections of *Cln2^R207X^* mice throughout disease progression for multiple markers of microglial activation (CD68) and astrogliosis. Thresholding image analysis revealed that localized CD68-positive microglial activation started within the primary motor (M1), somatosensory (S1BF), and visual (V1) cortices and the spinal cord ventral horn at 1 month (Figure 5, A and B). This increase in CD68 immunoreactivity was most pronounced within the somatosensory thalamocortical system, including S1BF and VPM/VPL, compared with other regions (Figure 5, A and B). The expression of disease-associated microglia (DAM) has been recently implicated in several neurodegenerative diseases (32–34). Therefore, we also stained *Cln2^R207X^* forebrain sections for DAM markers including major histocompatibility class II (MHCII) and C-type lectin domain containing 7a (Clec7a). MHCII-positive and Clec7a-positive microglia were observed in the VPM/VPL of *Cln2^R207X^* mice only at disease end stage (Supplemental Figure 3A).

**Figure 6:**
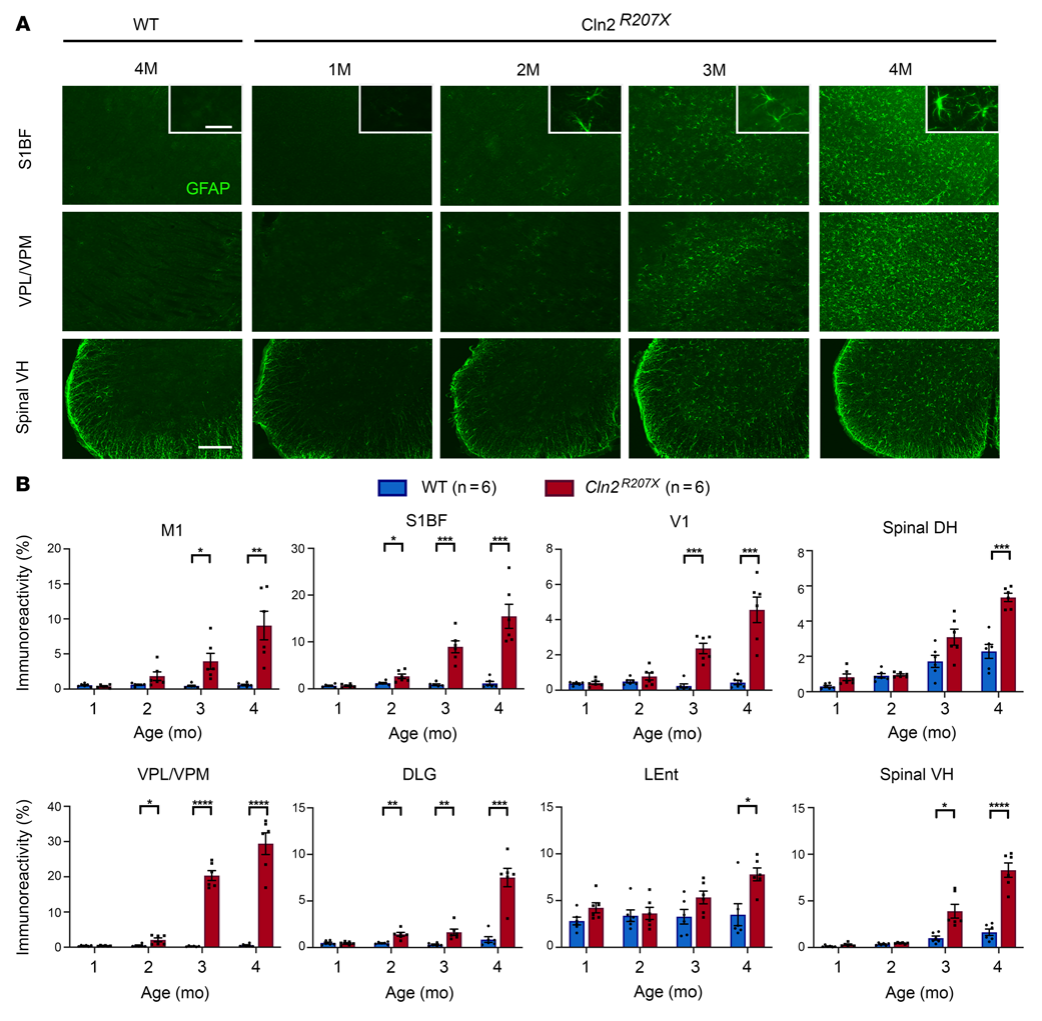
*Cln2^R207X^* mice show relatively delayed onset of astrogliosis. (**A**) Immunostaining for the astrocyte marker glial fibrillary acidic protein (GFAP, green) reveals the marked increase in the abundance of GFAP-positive cells and the intensity of GFAP immunoreactivity with disease progression in the primary somatosensory cortex (S1BF), ventral posterior nuclei of thalamus (VPM/VPL), and lumbar spinal ventral horn (VH) of *Cln2^R207X^* mice compared with WT control mice. Corresponding changes in astrocyte morphology are revealed in higher power insets. Scale bars: 200 μm, 20 μm (*insets*). (**B**) Quantitative thresholding image analysis of GFAP immunoreactivity verifies the progressive nature of astrogliosis at 12-, 24-, 36- and 48-week time points in the primary motor cortex (M1), S1BF, primary visual cortex (V1), VPM/VPL, dorsolateral geniculate nucleus (DLG), lateral entorhinal cortex (LEnt), lumbar spinal dorsal horn (DH), and lumbar spinal VH as early as 2 months in *Cln2^R207X^* mice (red bars) compared with age-matched WT control mice (blue bars). Dots represent values from individual animals. *P < 0.05, **P < 0.01, ***P < 0.001, ****P < 0.0001, multiple *t* test with Holm-Šídák correction. Values are shown as mean ± SEM (*n* = 6 mice per group).

Staining for glial fibrillary acidic protein (GFAP, astrogliosis marker) revealed an increase in GFAP immunoreactivity that was significant at 2 months in *Cln2^R207X^* brains and at 3 months of age in their spinal cords (Figure 6, A and B), indicating an onset of astrogliosis later than that of microglial activation. GFAP immunoreactivity was higher within S1BF and VPM/VPL than in other regions (Figure 6, A and B). Recently, the concept of neurotoxic versus neuroprotective (A1/A2) polarization of astrocytes has been proposed (35). Therefore, we investigated the expression of genes specific to A1 or A2 astrocytes in *Cln2^R207X^* brains by reverse transcription quantitative PCR (RT-qPCR). While serpin family G member 1 (A1-specific) and cardiotrophin-like cytokine factor 1 (A2-specific) were significantly elevated at 3 months of age, those genes were rather suppressed at 4 months (Supplemental Figure 3B). In contrast, complement component 3 and histocompatibility 2, D region locus 1 (A1-specific), and cluster of differentiation 14 (A2-specific) were significantly elevated (Supplemental Figure 3B).

### A distinct neuroinflammatory environment in Cln2^R207X^ mouse forebrains.

**Figure 7:**
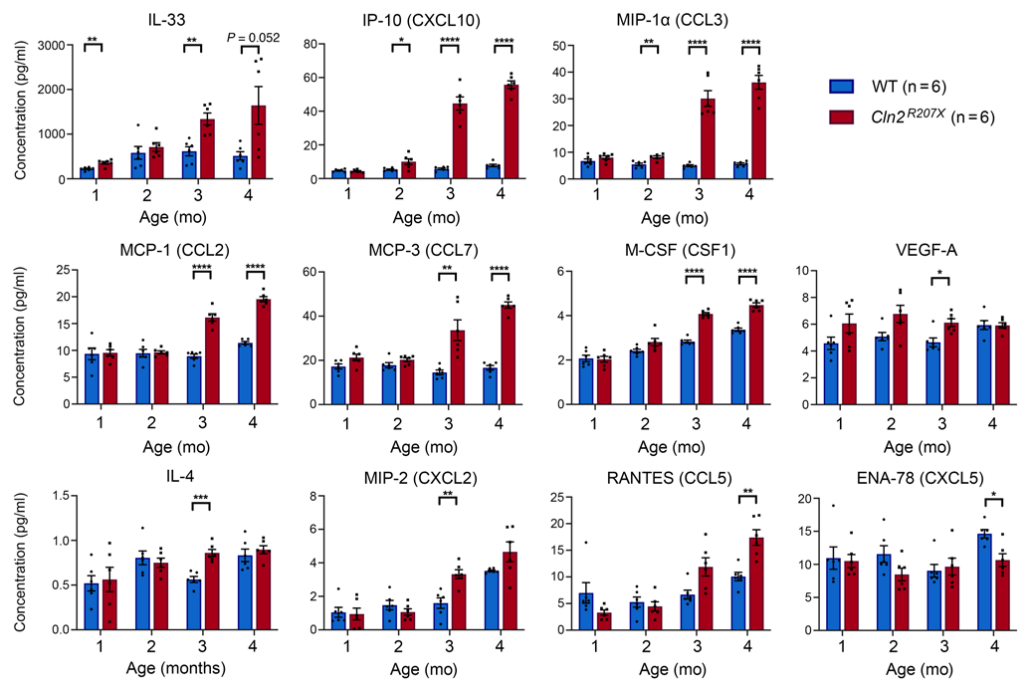
*Cln2^R207X^* mouse forebrains show distinct neuroinflammatory changes. Observed concentrations (pg/mL) of multiple cytokines and chemokines measured in forebrain homogenates reveal significant elevation of IL-33 at 1 month; IP-10 (CXCL10) and MIP-1α (CCL3) at 2 months; MCP-1 (CCL2), MCP-3 (CCL7), M-CSF (CSF1), VEGF-A, IL-4, and MIP-2 (CXCL2) at 3 months; and VEGF-A (CCL5) at 4 months and significant reduction of ENA-78 (CXCL5) at 4 months in *Cln2^R207X^* forebrains (red bars) compared with age-matched WT controls (blue bars). Dots represent values from individual animals. *P < 0.05, **P < 0.01, ***P < 0.001, ****P < 0.0001, multiple *t* test with Holm-Šídák correction. Values are shown as mean ± SEM (*n* = 6 mice per group). See Supporting Data Values for complete data.

We further investigated the nature of the neuroimmune response in *Cln2^R207X^* forebrains by measuring a panel of 23 chemokines and cytokines associated with inflammatory changes using an Affymetrix multiplex assay. There was a significant increase in IL-33 in *Cln2^R207X^* mice starting at 1 month of age and significant increases in interferon-γ–induced protein 10 (IP-10, or CXCL10) and macrophage inflammatory protein-1α (MIP-1α, or CCL3) starting at 2 months of age compared with WT controls (Figure 7). Increases in monocyte chemoattractant protein-1 (MCP-1, or CCL2), monocyte chemoattractant protein-3 (MCP-3, or CCL7), macrophage colony-stimulating factor (M-CSF, CSF1), VEGF-A, IL-4, and macrophage inflammatory protein 2 (MIP-2, or CXCL2) were observed in *Cln2^R207X^* mice at 3 months. Finally, a significant increase in RANTES (or CCL5), and a decrease in epithelial neutrophil activating peptide (ENA-78, or CXCL5), were observed in *Cln2^R207X^* mice at 4 months (Figure 7). There were no significant changes in a number of pro-inflammatory and antiinflammatory molecules, including IL-1α/β, TNF-α, IFN-γ, IFN-α, IFN-β, IL-2, IL-6, IL-10, CXCL1, and C-reactive protein (CRP), at any time point (Supplemental Figure 4). These data provide further evidence of a distinct neuroinflammatory environment in *Cln2^R207X^* mice, unlike that seen in other NCL or neurodegenerative models.

### AAV9-mediated gene therapy ameliorates neuropathological changes, markedly reduces spontaneous seizures, and prolongs the life span of Cln2^R207X^ mice.

**Figure 8:**
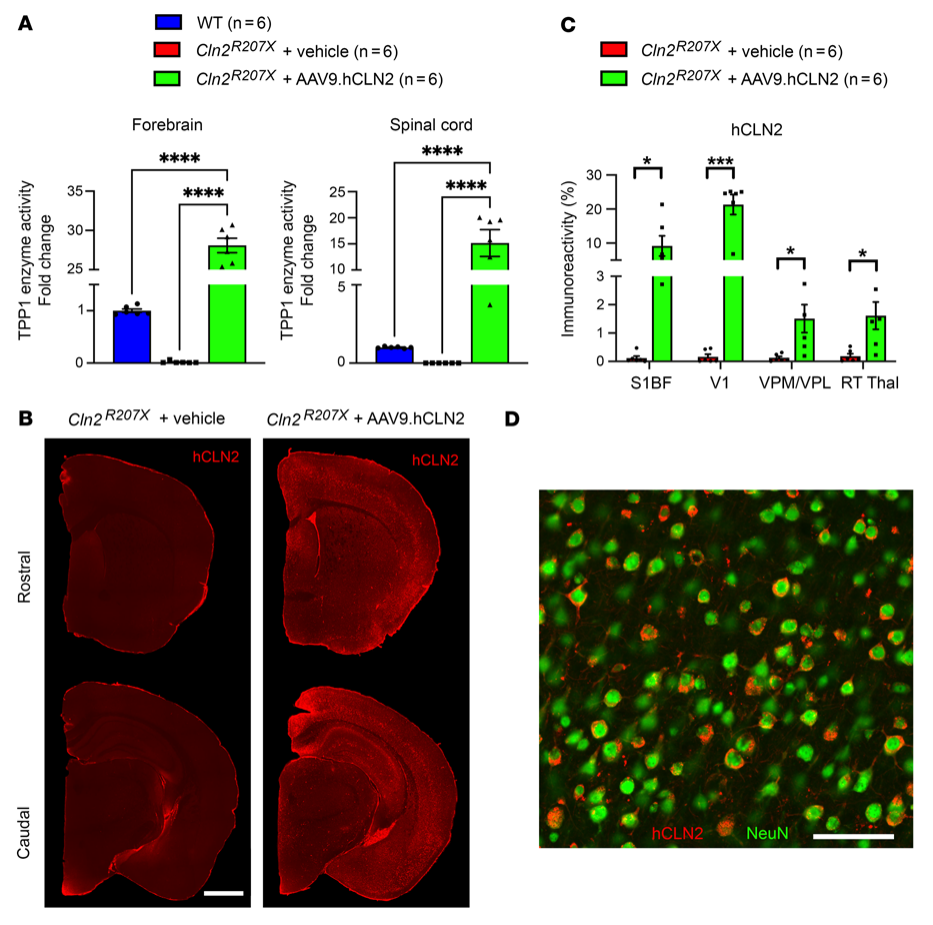
AAV9-mediated gene therapy rescues TPP1 activity in the central nervous system of *Cln2^R207X^* mice. (**A**) Tripeptidyl peptidase 1 (TPP1) activity assays reveal 28- and 15-fold average increases in TPP1 activity in the forebrain and spinal cord of AAV9.hCLN2-treated *Cln2^R207X^* mice compared with untreated WT controls at 3 months. *n* = 6 mice per group. (**B**) Immunostaining for human TPP1 (hCLN2, red) shows widespread distribution of AAV9-delivered hCLN2 in the forebrain of *Cln2^R207X^* mice. Coronal sections at equivalent levels are shown from vehicle-treated *Cln2^R207X^* (left) and AAV9-treated *Cln2^R207X^* forebrains (right). Scale bar: 1 mm. (**C**) Quantitative thresholding image analysis of hCLN2 reactivity reveals a significantly higher expression of AAV9-delivered hCLN2 across primary somatosensory cortex (S1BF), primary visual cortex (V1), ventral posterior nuclei of thalamus (VPM/VPL), and reticular nucleus of thalamus (Rt) in AAV9-treated *Cln2^R207X^* compared with those in vehicle-treated *Cln2^R207X^* mice. *n* = 6. Dots represent values from individual animals. Values are shown as mean ± SEM. *P < 0.05, ***P < 0.001, ****P < 0.0001, 1-way ANOVA with Bonferroni’s correction (**A**) and multiple *t* test with Holm-Šídák correction (**C**). (**D**) Co-immunostaining for NeuN (green) and hCLN2 (red) verifies that AAV9-delivered hCLN2 is predominantly expressed by neurons. Scale bar: 100 μm.

To test whether CLN2 gene delivery can treat these identified phenotypes in *Cln2^R207X^* mice, we administered a single ICV injection at P1 of either vehicle or 1 × 10^11^ genome copies (gc)/animal of an AAV9.hCLN2 vector that has recently been described (36). TPP1 enzyme assays at 3 months verified 28- and 15-fold average increases in TPP1 activity in the forebrain and spinal cord, respectively, of treated *Cln2^R207X^* mice compared with untreated WT controls (Figure 8A). Immunostaining showed widespread expression of hCLN2 in AAV9.hCLN2-treated *Cln2^R207X^* forebrains, with higher expression in cortical regions including S1BF and V1, particularly in large pyramidal neurons within layer V, than in subcortical regions including VPM/VPL and Rt (Figure 8, B and C). Counterstaining with a neuron-specific marker, NeuN, showed this hCLN2 transgene was expressed predominantly by neurons (Figure 8D).

**Figure 9:**
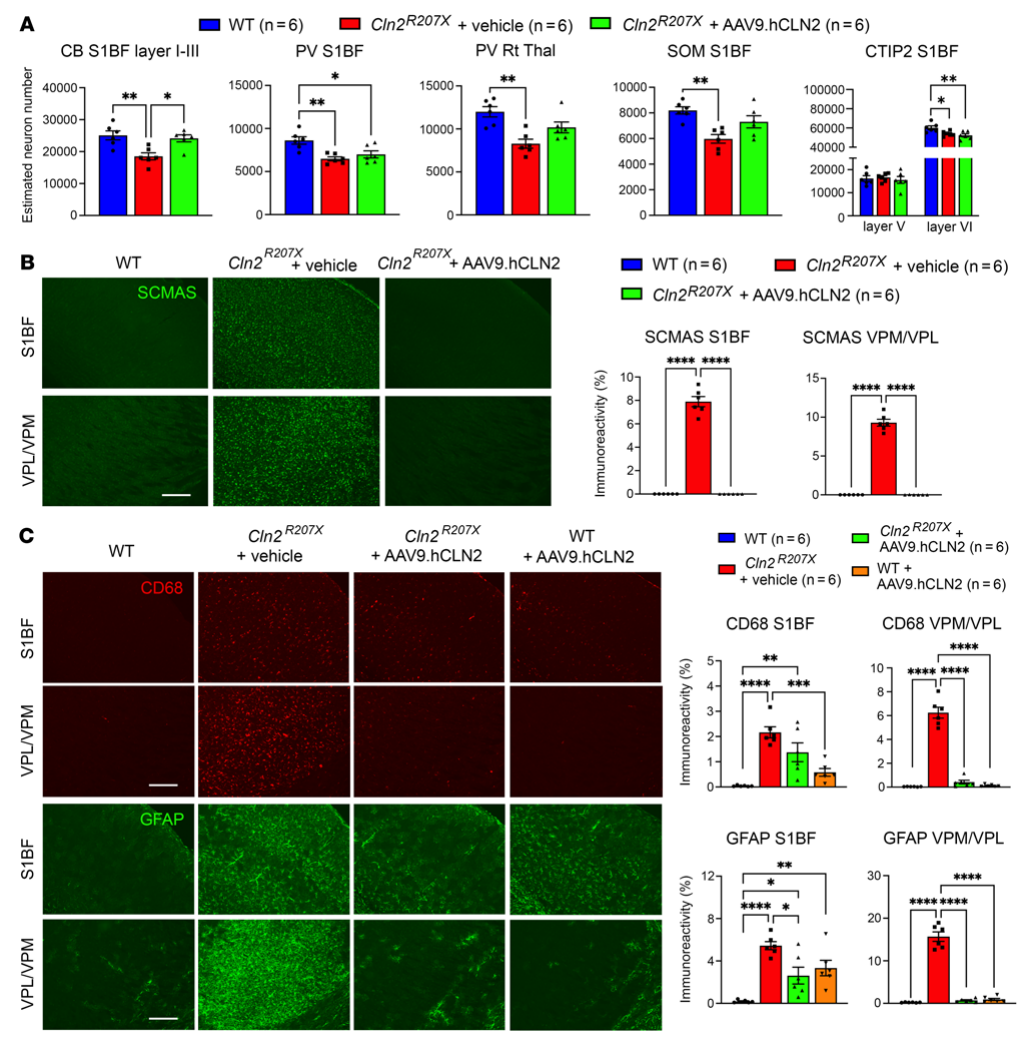
AAV9-mediated gene therapy attenuates neuropathological changes in *Cln2^R207X^* mice. (**A**) Unbiased stereological counts of immunostained neuron populations reveal significant prevention of calbindin-positive (CB) neuron loss within primary somatosensory cortex (S1BF), and a positive protective effect on parvalbumin-positive (PV) neurons within the thalamic reticular nucleus–positive (Rt) and somatostatin-positive (SOM) neurons within S1BF of AAV9.hCLN2-treated *Cln2^R207X^* mice, though these effects were not statistically significant. There was no significant treatment effect of AAV9 delivery of hCLN2 upon PV-positive neuron loss or transcription factor COUP TF1-interacting protein 2–positive (CTIP2) neurons within S1BF of *Cln2^R207X^* mice. (**B** and **C**) Immunostaining for subunit c of mitochondrial ATP synthase (SCMAS, green), cluster of differentiation 68 (CD68, red), and glial fibrillary acidic protein (GFAP, green) and quantitative thresholding image analysis on immunoreactivity of these markers reveal the effects of neonatal AAV9.hCLN2-treatment in *Cln2^R207X^* mice at 3 months. These include complete abrogation of SCMAS accumulation within both S1BF and ventral posterior nuclei of thalamus (VPM/VPL), complete prevention of microglial activation/astrogliosis within VPM/VPL, partial prevention of microglial activation within S1BF, and no significant change for astrogliosis within S1BF of AAV9.hCLN2-treated *Cln2^R207X^* mice at 3 months. Scale bar: 200 μm. Dots represent values from individual animals. Values are shown as mean ± SEM (*n* = 6 mice per group). *P < 0.05, **P < 0.01, ***P < 0.001, ****P < 0.0001, 1-way ANOVA with Bonferroni’s correction.

Histological analysis (*n* = 6 WT, *n* = 6 vehicle-treated *Cln2^R207X^*, and *n* = 6 AAV9.hCLN2-treated *Cln2^R207X^* mice) at 3 months revealed significant prevention of CB-positive neuron loss within S1BF, and an apparent protective effect on PV-positive neurons within Rt and SOM-positive neurons within S1BF, though these effects were not statistically significant (Figure 9A). To investigate whether the effects of AAV9.hCLN2 treatment also extended to immunohistochemically identified excitatory cortical neurons, we stained sections from 3-month-old WT and vehicle- and AAV9.hCLN2-treated *Cln2^R207X^* mice for the transcription factor COUP TF1-interacting protein 2 (CTIP2), which is predominantly expressed in layers V and VI of the cortex (37, 38). Unbiased stereological counts of the number of CTIP2-positive neurons revealed layer-specific treatment effects. Compared to WT mice there was no difference in the number of CTIP2-positive neurons in layer V of vehicle- and AAV9.hCLN2-treated *Cln2^R207X^* mice, but there were significantly fewer of these neurons in layer VI of S1BF (Figure 9A). However, this loss of CTIP2-positive layer VI neurons was not protected by AAV9.hCLN2 treatment.

Immunostaining for SCMAS revealed that storage material accumulation in *Cln2^R207X^* mice was completely abrogated by AAV9 gene therapy across all forebrain regions (Figure 9B and Supplemental Figure 5B). Immunostaining for CD68 showed less microglial activation within VPM/VPL of AAV9.hCLN2-treated *Cln2^R207X^* mice, but this treatment effect was less evident within S1BF, where microglial activation was still apparent (Figure 9C). Similarly, immunostaining for GFAP showed complete prevention of astrogliosis within VPM/VPL of AAV9.hCLN2-treated *Cln2^R207X^* mice and a partial, but statistically significant, decrease within S1BF in these mice (Figure 9C). We observed an unexpected increase in both CD68 and GFAP immunoreactivity within V1 of AAV9.hCLN2-treated *Cln2^R207X^* mice, though this immune response was not accompanied by significant loss of any neuron populations within this cortical region, including Nissl-stained pyramidal neurons and CB-, PV-, and SOM-positive interneurons (Supplemental Figure 5, A and C). To further investigate the possible cause of this unexpected neuroimmune response in the cortex of AAV9.hCLN2-treated *Cln2^R207X^* mice, we also treated neonatal WT mice via an ICV injection of the same AAV9.hCLN2 vector. Histological analysis at 3 months revealed a comparable increase of CD68 and GFAP immunoreactivity that was restricted to cortical regions of these AAV9.hCLN2-treated WT mice (Figure 9C and Supplemental Figure 5C), suggesting that these region-specific neuroimmune responses may be related either to the vector or the high level of TPP1 expression.

**Figure 10:**
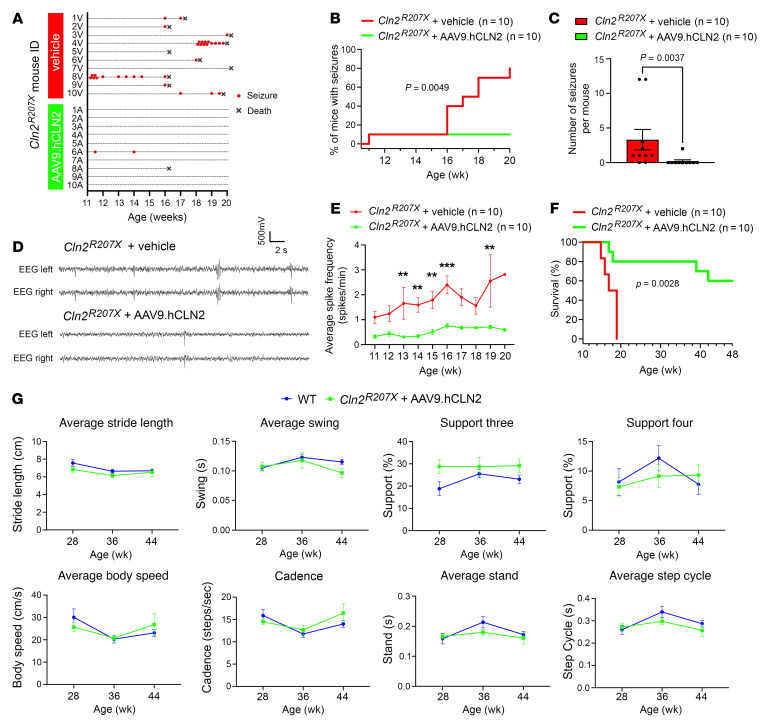
AAV9-mediated gene therapy prevents spontaneous seizures and extends the life span of *Cln2^R207X^* mice. (**A**) Time course of seizures (red dots) and deaths (black *X*s) in vehicle- and AAV9.hCLN2-treated mice. EEG recording reveals that the AAV9.hCLN2 treatment results in a significantly lower percentage of *Cln2^R207X^* mice who develop spontaneous seizures (**B**) and a significantly reduced total number of spontaneous seizures (**C**). *n* = 10 mice per group. (**D**) Representative EEG traces from vehicle- and AAV9.hCLN2-treated *Cln2^R207X^* mice at 16 weeks of age. (**E**) EEG recordings reveal that AAV9.hCLN2 treatment results in a significantly lower average spike frequency at 13, 14, 15, 16, and 19 weeks. *n* = 10 mice per group. (**F**) Survival studies out to 48 weeks reveal a significantly extended life span of AAV9.hCLN2-treated *Cln2^R207X^* mice (*n* = 10) compared with vehicle-treated *Cln2^R207X^* mice (*n* = 6). (**G**) CatWalk XT gait analysis reveals no significant difference between gait performance of AAV9.hCLN2-treated *Cln2^R207X^* mice at 28 (*n* = 8), 36 (*n* = 7), or 44 weeks (*n* = 6) and age-matched WT mice (*n* = 8 for all time points). **P < 0.01, ***P < 0.001. Log-rank (Mantel-Cox) test (**B** and **F**), Mann-Whitney *U* test (**C**), and 2-way ANOVA with Bonferroni’s correction (**E** and **G**). Dots represent values from individual animals, and values are shown as mean ± SEM (**C**, **E**, and **G**).

Long-term EEG monitoring revealed that significantly fewer treated mutant mice developed spontaneous seizures (Figure 10, A and B), and there was a marked reduction in the total number of spontaneous seizures (Figure 10C) in AAV9.hCLN2-treated versus vehicle-treated *Cln2^R207X^* mice. Only a single *Cln2^R207X^* mouse out of the 10 mice in the AAV9.hCLN2-treated group developed seizures (compared with 80% of the vehicle-treated *Cln2^R207X^* mice), and even this individual mouse that showed seizure activity of similar duration to untreated *Cln2^R207X^* mice did not die during an EEG monitoring period of up to 20 weeks (Figure 10A). Compared with vehicle-treated *Cln2^R207X^* mice there was a significant reduction in the frequency of interictal spikes in AAV9.hCLN2-treated *Cln2^R207X^* mice that was sustained between 11 and 20 weeks (Figure 10, D and E). A separate survival cohort (*n* = 6 vehicle-treated *Cln2^R207X^* mice and *n* = 10 AAV9.hCLN2-treated *Cln2^R207X^* mice) was monitored up to 48 weeks and demonstrated a significant extension of life span by AAV9.hCLN2 gene therapy (Figure 10F). Gait analysis of these AAV9.hCLN2-treated *Cln2^R207X^* mice at 28, 36, and 44 weeks revealed that all the gait parameters that were observed as abnormal in untreated *Cln2^R207X^* mice at 4 months (Figure 2) were not statistically different in AAV9.hCLN2-treated *Cln2^R207X^* mice from age-matched WT mice (Figure 10G).

## Discussion

CLN2 disease research has predominantly focused on the development of strategies to supply the missing TPP1 enzyme, which led to the approval of ERT with cerliponase alfa in the United States and Europe in 2017 (8). Although ERT slows CLN2 disease progression (9), cerliponase alfa is not curative and requires biweekly lifelong administration. Although the efficacy of AAV-mediated gene therapy using other serotypes has been tested previously in mice (21, 39–42), these studies could not assess seizure activity, as this had not been explored in any detail before the present study. In a clinical trial, an AAV serotype rh.10 vector expressing TPP1 slowed disease progression to an extent, although recombinant TPP1 therapy was more effective against neurological decline (43). To improve the therapeutic efficacy of gene therapy approaches, it will be important to learn more about CLN2 disease pathophysiology at both neuroanatomical and cellular levels. In this study, we have adopted a multidisciplinary approach that has systematically defined a potentially novel, robust, and quantifiable spontaneous seizure phenotype and also revealed a distinct staging of the underlying neuropathology in the forebrain and spinal cord of *Cln2^R207X^* mice. Our mouse data also show a single neonatal ICV injection of AAV9.hCLN2 vector can serve as a viable preclinical gene therapy strategy. Administration of this vector has a significant therapeutic impact on seizure occurrence, gait performance, and life span in *Cln2^R207X^* mice, together with ameliorating, and in some cases completely preventing, many of the associated neuropathological changes.

Although epileptic seizures are a key presenting symptom in CLN2 patients throughout their disease course (5–7), no previous study has investigated the seizure phenotype of any animal model of CLN2 disease. The EEG background abnormalities and pronounced spontaneous seizure phenotypes in *Cln2^R207X^* mice revealed by our longitudinal EEG recording are progressive in nature and comparable to those in mice modeling infantile NCL or CLN1 disease (*Cln1^–/–^* mice) and other genetic epilepsy models, such as tuberous sclerosis complex 1 mice (13, 44, 45). The majority of *Cln2^R207X^* mice died shortly after seizures, and it will be important to determine whether these events may be related. Currently, we cannot definitively conclude that seizure activity and the associated fatality are causally related to cortical neuron loss. It will be important to mechanistically address this issue and explore how seizure activity, interneuron network activity, and the loss of interneuron and other cortical neuron populations may be related. As seizure duration did not significantly change during disease progression, there does not appear to be a direct relationship between seizure duration and seizure-related mortality. We can only speculate why *Cln2^R207X^* mice apparently die shortly after seizures of relatively short duration. However, our findings are consistent with the previous report of sudden premature death following seizures of relatively short duration (<2 minutes) in a mouse model of Dravet syndrome, another pediatric-onset genetic epileptic disorder (46). It will be important to determine if the cause of death in our *Cln2^R207X^* mice is related to the mechanisms of sudden unexplained death in epilepsy (SUDEP) (47), but this lies beyond the scope of this paper. However, anecdotally in patients, even short tonic-clonic seizures can be associated with cardiorespiratory instability and SUDEP (48).

Our EEG findings provide a potentially novel, quantifiable, and clinically relevant behavioral phenotype in *Cln2^R207X^* mice that we have demonstrated can serve as a robust readout of efficacy in preclinical therapeutic studies. Indeed, our data reveal AAV9.hCLN2 treatment almost completely prevents seizures in *Cln2^R207X^* mice, and significantly reduces interictal epileptiform activity (Figure 10E), in addition to significantly extending their life span (Figure 10F). Given that seizure activity and death are closely associated in *Cln2^R207X^* mice, this extended life span may be due to preventing these seizures, but treatment effects on other pathological consequences of TPP1 deficiency cannot be excluded.

Gait abnormalities in *Cln2^R207X^* mice appeared only toward disease end stage and to a remarkably lesser extent than those documented in *Cln1^–/–^* mice (19). This may be related to the less severe and delayed onset of spinal cord pathology in *Cln2^R207X^* versus *Cln1^–/–^* mice but may also be due to the effects of TPP1 deficiency on other brain regions. These data are also unexpected given that severe gait decline is seen in patients with CLN2 disease (49). Untreated *Cln2^R207X^* mice may die because of seizure-related fatality, before their gait abnormalities become severe enough to prevent them from accessing food and water. Nevertheless, our data also reveal that AAV9.hCLN2 treatment is able to prevent these apparently fatal seizures and reduce interictal activity in *Cln2^R207X^* mice, significantly extending their life span. These surviving treated mice now lived out to 48 weeks of age, and their gait performance was almost completely preserved and was predominantly indistinguishable from WT mice, even at nearly 3 times the age when untreated *Cln2^R207X^* mice typically died.

Another marked difference from other forms of NCL is the timing of the regional specificity of neuropathology in *Cln2^R207X^* mice. Our data for both Nissl-stained and immunostained neurons reveal the first detectable neuron loss to be in the cortex before subsequently occurring in the thalamus of *Cln2^R207X^* mice. This is in the opposite order in which these brain regions are affected in other forms of NCL, where the loss of thalamic relay neurons precedes neuron loss in the corresponding cortical target region (12–17). Similarly, in contrast with the early involvement of the spinal cord compared to the forebrain in CLN1 mice (18, 19), we found that spinal pathology in *Cln2^R207X^* mice started only after brain pathology was present. Moreover, unlike CLN1 disease or other epilepsy models (50–52), there were no significant loss of Nissl-stained neurons and only very limited interneuron loss in the hippocampus of *Cln2^R207X^* mice.

Defining seizure phenotypes in *Cln2^R207X^* mice guided us to extend our pathological characterization of these mice to explore potential GABAergic interneuron deficits, which are implicated as contributing to both neurodegenerative diseases and epilepsy (26, 27, 53). Our data reveal cortical interneurons are severely affected in *Cln2^R207X^* mice. In contrast, only very limited hippocampal interneuron loss was apparent, even at disease end stage. Within the cortex, neuron populations expressing different calcium-binding proteins were affected similarly in *Cln2^R207X^* mice and were consistently more severely affected in layers II and III than in deeper cortical layers. It is important to highlight that CB expression is not exclusive to interneurons but is also present in populations of excitatory neurons in both the hippocampus and cortex (54, 55). Our CTIP2 data reinforce that certain cortical excitatory neurons are also affected by TPP1 deficiency, albeit in a layer-specific fashion. However, cortical CB-, CR-, PV-, or SOM-positive neuron loss invariably preceded that of Nissl-stained neurons in the same cortical region or of PV-positive interneurons in the thalamus. If these treated *Cln2^R207X^* mice do not die from the apparently fatal seizures that we have documented, they may go on to display more severe pathology that more closely resembles that seen in both the cortex and hippocampus of human patients with CLN2. This includes the loss of both interneuron and excitatory neurons in different subfields of the hippocampus and widespread regions of the cortex, along with associated changes in astrocytosis and microglial activation (56, 57).

Although our EEG monitoring cannot precisely locate the anatomical source of seizure activity, our histological data suggest a cortical rather than hippocampal origin for seizures in *Cln2^R207X^* mice. However, we cannot exclude the possibility that pathological effects in other brain regions may also be involved in seizure generation in these mice. Nevertheless, it is surprising that there was no significant change in the overall concentration of cortical neurotransmitters, including GABA. This may reflect possible compensatory mechanisms to maintain total cortical GABA levels or the inability of our analysis to distinguish between intracellular and extracellular levels of these cortical neurotransmitters.

Our comprehensive neuropathological characterization revealed a profound accumulation of SCMAS across CNS regions in *Cln2^R207X^* mice present as early as P1. Although storage material accumulation does not necessarily appear to be toxic (10, 58), storage burden serves as a useful quantitative outcome measure for assessing therapeutic efficacy and appeared to be completely prevented in widespread brain regions by neonatal AAV9.hCLN2 gene therapy. Early localized activation of both microglia and astrocytes that precedes subsequent neuron loss is also a neuropathological hallmark across multiple forms of NCL (10, 11). Progressive microglial activation and astrogliosis are also present in *Cln2^R207X^* mice and are most pronounced in the sensory corticothalamic system compared with other CNS regions. However, unlike other forms of NCL, early-onset microglial activation in *Cln2^R207X^* mice did not coincide with astrocytosis (1–2 months depending on the region). Instead, astrogliosis was relatively delayed and paralleled or immediately preceded the onset of Nissl-stained neurons loss 1 month later. Both microglial activation and astrogliosis were largely prevented in the thalamus of AAV9.hCLN2-treated *Cln2^R207X^* mice, but the treatment effect was less pronounced in the cortex of these mice, where glial activation was more pronounced.

We found a delay of several months between the early onset of CD68-positive microglial activation and the late onset of elevated staining for the DAM markers MHCII and Clec7a in *Cln2^R207X^* brains. In addition, our RT-qPCR evidence for changes in the expression of only a subset of A1/A2-specific astrocytic markers is in stark contrast to the pronounced typical A1-specific molecular signature transformation that occurs in *Cln1^–/–^* mice (59). Although the contribution of the heterogeneous subtypes of reactive astrocytes to neurodegeneration remains unclear, such data highlight that astrogliosis in CLN2 disease mice differs at a molecular and morphological level compared with that in CLN1 disease. Compared with our previous data from *Cln1^–/–^* mice (19), we also found changes in the expression of only a restricted subset of cytokines and chemokines in the forebrain of *Cln2^R207X^* mice. Indeed, in these mice, there were no changes in the expression of multiple cytokines and chemokines, including IFN-γ and IL-10, that are significantly altered in *Cln1^–/–^* mice (19). This lack of altered expression of these central pro-inflammatory and antiinflammatory molecules implies that there may be a lesser neuroinflammatory component in CLN2 disease than in CLN1 disease. Taken together, our results reveal a potentially novel regional pattern of neuropathological progression in CLN2 disease mice that is in stark contrast to other forms of NCL. This is especially the case compared with CLN1 disease mice, where the spinal cord, thalamus, and cortex are affected in the opposite sequence (18, 19).

Utilizing this defined seizure phenotype and these neuropathological changes as robust readouts, this preclinical study has shown the efficacy of an AAV9.hCLN2 gene delivery strategy for CLN2 disease. The safety and tolerability of AAV9.hCLN2 vector used in this study have previously been tested in both mice and cynomolgus monkeys (36). Quantitative TPP1 enzyme assays and immunostaining verified the widespread distribution of transgene expression across forebrains and spinal cords after a single neonatal ICV injection of the AAV9 vector. Notably, preferential expression of hCLN2 in cortical versus subcortical regions was apparent in treated mice. This regional hCLN2 expression pattern is in line with previous studies reporting the regional pattern of AAV9 vector transduction following neonatal ICV injection (60). Nevertheless, AAV9.hCLN2 gene delivery completely prevented SCMAS accumulation throughout the forebrain of *Cln2^R207X^* mice. In contrast, the therapeutic effect of AAV9.hCLN2 gene delivery on CD68-positive microglial activation and GFAP-positive astrogliosis appeared to be region dependent, with greater efficacy within the thalamus, where both forms of glial activation and the effects of TPP1 deficiency were less pronounced in untreated *Cln2^R207X^* mice. Our data suggest it is more challenging to completely treat pathological changes in the *Cln2^R207X^* cortex, and the higher level of human TPP1 enzyme expression may cause a neuroimmune response, as was also evident in the cortex of AAV9.hCLN2-treated WT mice. Similar immune responses to elevated enzyme expression have been reported in nonhuman primate brains (36, 61), although such treatment-related immunological reactions may be different in humans. However, there was no evidence that this moderate treatment-related neuroimmune response exacerbated or led to neuron loss. As such, the differences in the regional response to gene therapy are likely a result of several factors, including regional differences in the impact of TPP1 deficiency, in the distribution of AAV9.hCLN2 transduction, and in responses to elevated TPP1 expression.

Our data from untreated *Cln2^R207X^* mice reveal a pronounced impact of TPP1 deficiency upon multiple populations of cortical neurons. This is not exclusive to neurons expressing commonly used interneuron markers but also extends to excitatory neurons later in disease progression. Although AAV9.hCLN2 gene therapy almost completely prevented the occurrence of seizures in these mice, only CB-positive cortical neurons were significantly protected by this treatment, with almost no positive impact on PV-positive interneurons. This treatment effect did not extend to layer VI CTIP2-positive excitatory neurons, and this subpopulation-dependent treatment effect suggests that TPP1 deficiency may lead to irreversible cellular damage in certain neuron populations during early cortical development and that CB-positive cortical neurons may be most associated with seizure onset in *Cln2^R207X^* mice. Our data suggest this treatment effect of AAV9.hCLN2 gene therapy likely includes both inhibitory and excitatory neurons. It will be important to further investigate the nature of cortical neuron dysfunction and how this may contribute to epileptogenesis in CLN2 disease. An unexpected feature of our efficacy data is that AAV9.hCLN2 treatment produces significant effects upon seizure-related parameters, without completely preventing all neuropathological phenotypes. We hypothesize that elevated levels of vector-expressed TPP1 enzyme are sufficient to prevent whatever as-yet-unidentified, pathological changes or combination of events are responsible for seizure generation. Nevertheless, our data have important and immediate translational implications, as AAV9.hCLN2 gene delivery effectively prevented spontaneous seizures, significantly reduced interictal epileptiform activity, and significantly extended the life span of *Cln2^R207X^* mice (Figure 10). These data provide a rationale for the subsequent clinical testing of AAV9.hCLN2 in patients with CLN2 disease to determine its efficacy.

In conclusion, our data reveal phenotypes in *Cln2^R207X^* mice that differ markedly in their nature and timing compared with other mouse models of NCL. Of greatest clinical relevance, we have documented a potentially novel and robust seizure phenotype, which may be related to the pronounced loss of cortical neuron populations including interneurons. These data emphasize that while disease presentation may be broadly similar across various forms of NCL, this detailed characterization has revealed phenotypes in CLN2 disease mice that are likely unique in many fundamental aspects. Crucially, these data provide robust quantitative landmarks that are relevant to the human disease for judging therapeutic efficacy. We demonstrate that a single ICV injection of AAV9.hCLN2 vector effectively attenuates many of these defined neuropathological phenotypes, effectively prevents seizures, reduces interictal epileptiform activity, and significantly extends survival in *Cln2^R207X^* mice. During this extended survival period, treated mice also showed markedly improved gait performance, another test that is used clinically to assess disease progression in children with CLN2 disease (49). Given this robust preclinical efficacy and the documented safety of the AAV9.hCLN2 vector in a nonhuman primate, AAV9.hCLN2 gene therapy is now a candidate for further clinical investigation.

## Methods

### Mice.

*Cln2^R207X^* mice were first generated by Geraets et al. (23) (RRID: IMSR_JAX:030696, gift of Jill M. Weimer and David A. Pearce, Sanford Research, Sioux Falls, South Dakota, USA). *Cln2^R207X^* and WT (generated in-house from crosses of heterozygous *Cln2^R207X^* mice) mice were maintained separately on a congenic C57BL/6J background, housed in an animal facility at Washington University School of Medicine under a 12-hour light/12-hour dark cycle, and provided food and water ad libitum. To characterize disease-associated phenotypes, *n* = 6 mice of each genotype were used, except for gait analysis and EEG recording (*n* = 10) and RT-qPCR/neurotransmitter analysis (*n* = 4). Unless otherwise stated, an equal number of both males and females were included in each group.

### Tissue processing and Nissl staining.

Processing of both forebrain and lumbar spinal cord tissues and Nissl staining were performed, as described previously (18, 50, 62). See Supplemental Methods for further details.

### Immunohistochemistry and imaging.

A 1-in-6 series of coronal forebrain hemisections and a 1-in-48 series of 40 μm coronal spinal cord sections from each mouse were stained on slides using a modified immunofluorescence protocol using the TrueBlack Lipofuscin Autofluorescence Quencher (Biotium) (19, 63). See Supplemental Methods for further details, including primary and secondary antibodies. To visualize GABAergic interneurons for stereological counts, a 1-in-6 series of coronal hemisections were stained on slides using a modified immunoperoxidase protocol (18, 50). See Supplemental Methods for further details, including used antibodies. All images were taken on a Zeiss Axio Imager.Z1 microscope using StereoInvestigator (MBF Bioscience) software or a Zeiss LSM880 confocal laser scanning microscope with Airyscan and ZEN 2 software (blue edition, Zeiss).

### Quantitative thresholding image analysis.

To quantify storage material accumulation (SCMAS immunoreactivity) and glial activation (GFAP-positive astrocytes and CD68-, MHCII-, or Clec7a-positive microglia), semiautomated thresholding image analysis was performed as described previously (19, 63). This involved collecting slide-scanned images at original magnification, 10× (Zeiss Axio Scan.Z1 fluorescence slide scanner), from each animal. Contours of appropriate anatomical regions were then drawn, and images were subsequently analyzed using Image-Pro Premier (Media Cybernetics) using an appropriate threshold that selected the foreground immunoreactivity above the background. All thresholding data (GFAP, CD68, and SCMAS) were expressed as the percentage area within each anatomically defined region of interest that contained immunoreactivity above the set threshold for that antigen (% immunoreactivity).

### Stereological counts of neuron number.

Unbiased design–based optical fractionator counts of Nissl-stained neurons and GABAergic interneurons were performed using StereoInvestigator in a 1-in-6 series of forebrain hemisections and in a 1-in-48 series of spinal cord sections, as described previously (18, 19, 50). Specific sizes of sampling grid, counting frame, and appropriate objectives were used for each region of interest and type of staining, as detailed in the Supplemental Methods.

### Total RNA extraction, cDNA synthesis, and RT-qPCR.

Total RNA was extracted from forebrain homogenates from each animal and purified using TRIzol (Thermo Fisher Scientific), as previously described (64). Subsequently, cDNA was synthesized using Random Primers (Invitrogen) and SuperScript II Reverse Transcriptase (Invitrogen) according to the manufacturer’s protocol. All RT-qPCR reactions for each gene of interest were performed in triplicate. RT-qPCR was performed using SYBR Green PCR Master Mix (Applied Biosystems) with primers whose sequences are listed in the Supplemental Methods. The 2^−ΔΔCt^ of gene expression was analyzed for each gene and normalized with the reference gene GAPDH.

### Cytokine and chemokine profile analysis.

Cytokine and chemokine profiling in proteins extracted from forebrain homogenates was performed using an Affymetrix multiplex assay, as described previously (19). Customized Procartaplex Luminex assays, 24-plex, for CRP, ENA-78 (CXCL5), eotaxin (CCL11), growth-regulated oncogene α (GROα, or KC/CXCL1), IFN-α, IFN-β, IFN-γ, IL-1α, IL-1β, IL-2, IL-4, IL-6, IL-10, IL-33, IP-10 (CXCL10), MCP-1 (CCL2), MCP-3 (CCL7), M-CSF, MIP-1α (CCL3), MIP-1β (CCL4), MIP-2α (CXCL2), RANTES (CCL5), TNF-α, and VEGF-A were run in duplicate. See Supplemental Methods for further details.

### Quantitative gait analysis.

The CatWalk XT gait analysis system (Noldus Information Technology) was used to study gait performance at monthly intervals, as described previously (19, 65). See Supplemental Methods for further details.

### EEG monitoring.

WT and *Cln2^R207X^* mice underwent continuous video EEG monitoring starting at 10 weeks, using standard methods for implanting epidural electrodes and EEG recording under isoflurane anesthesia, as previously described (44, 66, 67). Burr holes for the frontal reference electrodes were made (anterior +0.5 mm, lateral ±0.5 mm; bregma) and secured via screws. Two bilateral “active” recording electrodes were placed over the parietal cortex (posterior –2.5 mm, lateral ±1.5; bregma), and a ground screw was secured over the cerebellum (posterior –6.2 mm, lateral ±0.5; bregma). At least 72 hours after recovering from surgery, groups of 4 mice were placed in individual cages and connected via a custom flexible cable attached to the exposed pin header for recording. Continuous bilateral cortical video EEG signals starting at 10 weeks were acquired using a referential montage using Stellate or LabChart (AdInstruments) acquisition software and amplifiers until *Cln2^R207X^* mice died or until 20 weeks in the case of WT mice. Signals were amplified at original magnification, 10,000×, with high-pass (0.5 Hz) and low-pass (100 Hz) filters applied. EEG signals were digitized at 250 Hz, and time-locked video EEG was collected continuously. Electrographic seizures were identified by their characteristic pattern of discrete periods of rhythmic spike discharges that evolved in frequency and amplitude, lasting at least 10 seconds, typically ending with repetitive burst discharges and voltage suppression. Interictal epileptiform spikes and bursts of spikes were defined as a single sharp/fast (<200 ms) discharge or a burst of sharp discharges that disrupted the typical electrographic background and were greater than 2.5× amplitude compared with the surrounding background rhythm. Interictal epileptiform spike analysis was performed while blinded by averaging the number of abnormal spikes and/or spike bursts in 1-minute epochs, every 2 hours for 1 day each week. Epochs with obvious visual artifacts were excluded.

### Neurotransmitter analysis.

The concentration of cortical neurotransmitters including glutamate (Glu), glutamine (Gln), and GABA was measured by LC-MS/MS. See Supplemental Methods for further details.

### AAV9-mediated gene therapy in Cln2^R207X^ mice.

All treated neonatal *Cln2^R207X^* mice in this study were injected into the lateral ventricle with 5 μL of vehicle or an AAV9.hCLN2 vector (1 × 10^11^ gc per mouse) that has previously been described (36). Briefly, AAV9.hCLN2 vector contained the codon-optimized human CLN2 cDNA sequence flanked by AAV2 inverted terminal repeats, and its expression was driven by a CB7 promoter, a hybrid between a cytomegalovirus immediate-early enhancer and the chicken β-actin promoter. Transcription from this promoter was enhanced by the presence of the chicken β-actin intron. The polyadenylation signal for the expression cassette was from the rabbit β-globin gene. The sequence of the human CLN2 cDNA and the parental plasmid used to generate our vector is included in Supplemental Data 1. In the current study, this AAV9.hCLN2 vector was administered at a single dose of 1 × 10^11^ gc per mouse. This dose was selected based on the previous pilot study in CLN2 mice with doses ranging from 1.25 × 10^10^ to 8.5 × 10^11^ gc/mouse administered by ICV injection to assess impact on life span, CNS pathology, and central and peripheral toxicology, with no treatment-related adverse in-life findings (68).

In the current study, at 3 months (disease end stage), both vehicle-treated and 1 × 10^11^ gc/mouse AAV9-treated *Cln2^R207X^* mice and untreated WT mice (*n* = 6 per group; 3 male and 3 female) were sacrificed and had collected tissue analyzed for TPP1 enzyme activity assays and histology. Long-term EEG recordings were performed on a separate cohort of vehicle-treated *Cln2^R207X^* (*n* = 10; 3 male and 7 female) and 1 × 10^11^ gc/mouse AAV9-treated *Cln2^R207X^* mice (*n* = 10; 5 male and 5 female) from 10.5 to 20 weeks. A separate survival cohort of vehicle-treated *Cln2^R207X^* (*n* = 6; 2 male and 4 female) and 1 × 10^11^ gc/mouse AAV9-treated *Cln2^R207X^* mice (*n* = 10; 4 male and 6 female) was monitored out to 48 weeks. All analyses were performed while blinded to genotype and treatment status.

### TPP1 enzyme activity assays.

TPP1 enzyme activity in proteins extracted from forebrain homogenates was performed using fluorometric assays, as described previously (23, 30). See Supplemental Methods for further details.

### Statistics.

All statistical analyses were performed using GraphPad Prism version 9.1.0 for MacOS (GraphPad Software). Two-tailed unpaired *t* test or Mann-Whitney *U* test was used for comparison between 2 groups based on distributions of data. A multiple *t* test with a post hoc Holm-Šídák correction was used for histological data at multiple time points and cytokine and chemokine profiling data. A 1-way ANOVA with a post hoc Bonferroni’s correction was used for comparison between 3 groups or more, and a 2-way ANOVA with a post hoc Bonferroni’s correction was used for gait analysis. Log-rank (Mantel-Cox) test was used for the onset of spontaneous seizures and survival studies. A *P* ≤ 0.05 was considered significant.

### Study approval.

All animal procedures were performed in accordance with NIH guidelines under protocols 2018-0215 and 21-0292 approved by the IACUC at Washington University School of Medicine.

### Data availability.

All data are available in the main text or the supplemental materials (see Supporting Data Values).

## Author contributions

JDC, KT, NB, MW, and MSS conceived and designed the study. KT carried out the pathology experiments, tissue culture experiments, RT-qPCR, and statistical analysis on results from all experiments; EME performed gait analysis; SHW assisted with immunohistochemistry; and NRR performed EEG recording. HRN participated in mouse sample preparation and contributed to interpretation of histological and gait analysis data. JTD contributed to interpretation of gait analysis and seizure data. NB and OD contributed to production of an AAV9.hCLN2 vector. KT and JDC wrote the manuscript with input from all the authors. All authors read and approved the final manuscript.

## Supplementary Material

Supplemental data

Supplemental data set 1

Supplemental video 1

Supporting data values

## Figures and Tables

**Table 1:**
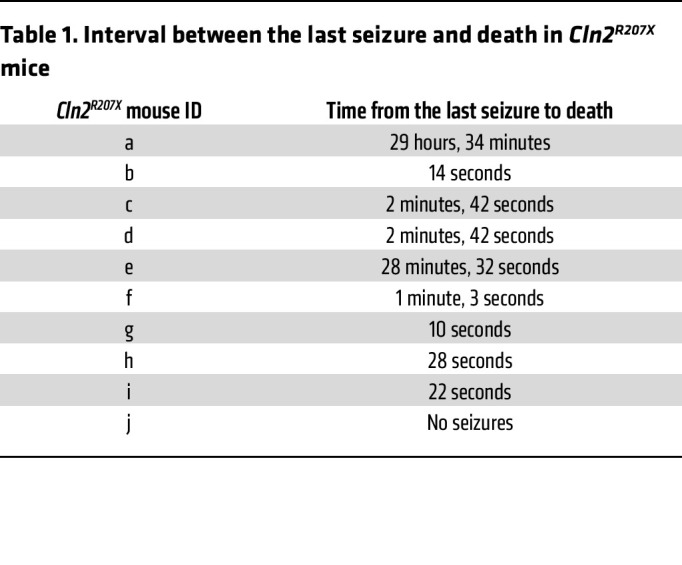
Interval between the last seizure and death in *Cln2^R207X^* mice

## Version 1 04/27/2023

In-Press Preview

## Version 2 06/15/2023

Electronic publication
