# Supplementary material for: Gene therapy ameliorates spontaneous seizures associated with cortical neuron loss in a Cln2R207X mouse model
Source: J Clin Invest. 2023 Jun 15;133(12):e165908. doi: 10.1172/JCI165908 (PMC10266778; doi:10.1172/JCI165908)
Supplement: Supplemental data [file jci-133-165908-s012.pdf]

Supplemental Materials and Methods

Tissue processing and Nissl staining

Mice were anesthetized using 2% isoflurane anesthesia and transcardially perfused with PBS. The brain and spinal column were dissected out. The brain was bisected in the sagittal plane and spinal column divided into cervical, thoracic and lumbosacral segments. One brain hemisphere and the thoracic segment of cord were frozen and stored at -80°C for biochemical and molecular analysis, while the other brain hemisphere and cervical and lumbo-sacral segments of cord were fixed in 4% paraformaldehyde (PFA) solution for 48 hours and subsequently cryoprotected in 30% sucrose in 50 mM TBS (pH=7.6). Cryoprotected brain and spinal cord tissues were sectioned along the coronal axis at 40µm using a Microm HM430 freezing microtome (Microm International, Germany) equipped with a Physitemp BFS-40MPA freezing stage (Physitemp, Clifton, NJ) prior to histological analysis.

40 µm brain and spinal cord sections were stained for cresyl fast violet (Nissl) to reveal their cytoarchitecture. For mouse tissue, a 1 in 6 series of brain and 1 in 48 series of spinal cord sections were mounted on chrome-gelatin coated microscope slides (ThermoFisher Scientific) and air-dried overnight. All sections were then incubated for at least 45 minutes at 60°C in 0.1% cresyl fast violet and 0.05% acetic acid. Stained sections were then differentiated through a series of graded ethanol solutions (70%, 80%, 90%, 95% and 2x100%) (Fisher Scientific, MA) before clearing in Xylene (Fisher Scientific) and coverslipping with DPX (Fisher Scientific), a xylene-based mountant.

Unbiased stereological counts of neuron number

Nissl-stained neurons with a clearly identifiable nucleus were counted using the optical fractionator method with the following sampling scheme using 100× oil objective (NA 1.4). Counting frame for S1BF layer V, V1 layer V, VPM/VPL, spinal cord ventral horns, and spinal cord dorsal horn layer III-V, 90 × 100 µm; counting frame for hippocampal CA1, 80 × 60 µm; sampling grid for S1BF layer V and V1 layer V, 450 × 450 µm; sampling grid for VPM/VPL, 600 × 600 µm; sampling grid for spinal cord ventral horn and dorsal horn layer III-V, 350 × 350 µm; sampling grid for hippocampal CA1, 250 × 250 µm.

Neurons expressing calbindin (CB), parvalbumin (PV), somatostatin (SOM), calretinin (CR), and Coup-TFI interacting protein 2 (CTIP2) with clearly identifiable immunoreactivity were counted using the optical fractionator method with the following sampling scheme and objective. CB in S1BF layer I-III and V1 layer I-III, counting frame 200 × 180 µm, sampling grid 600 × 500 µm, objective 40× oil (NA 1.30); PV and SOM in S1BF and V1, counting frame 400 × 360 µm, sampling grid 800 × 800 µm, objective 20×; PV in Rt of thalamus, counting frame 100 × 120 µm, sampling grid 300 × 400 µm, objective 63× oil (NA 1.4); CTIP2 in S1BF layer V, counting frame 80 × 60 µm, sampling grid 300 × 200 µm, objective 100× oil (NA 1.4); CTIP2 in S1BF layer VI, counting frame 80 × 60 µm, sampling grid 350 × 350 µm, objective 100× oil (NA 1.4). CR in cortical regions and all interneurons in hippocampal regions were counted manually.

Immunohistochemistry

<u>A modified immunofluorescence protocol</u>: a one-in-six series of coronal forebrain sections and a one-in-forty-eight series of coronal spinal cord sections were mounted on 25 x 75 mm microscope slides (Fisher Scientific) and air-dried for 30 minutes before blocking in Tris-

buffered saline (TBS) with 4% Triton X-100 (Alfa Aesar) and 15% normal goat serum (Vector) for 1 hour in room temperature. Sections were then incubated for the following primary antibodies in TBS with 4% Triton X-100 and 10% normal goat serum for 2 hours: rabbit anti-GFAP 1:1000, DAKO #Z0334, rat anti-CD68 1:400, Bio-Rad #MCA1957, rabbit anti-SCMAS 1:400, abcam #ab181243, rat anti-MHC-II 1:200, Invitrogen #14-5321-82, rat anti-Clec7a 1:200, InvivoGen #mabg-mdect, rabbit anti-parvalbumin (PV) 1:500, SWant #PV27a, mouse anti-calbindin (CB) 1:1000, SWant #CB300, mouse anti-GAD67 1:200, EMD Millipore #MAB5406, rabbit anti-NeuN 1:800, abcam #ab177487, mouse anti-hCLN2 1:800, EMD Millipore #MABN1806, rabbit anti-GABA 1:500, Sigma #A2052. After 3 times of washing with TBS, sections were incubated for the following secondary antibodies in TBS with 4% Triton X-100 and 10% normal goat serum for 2 hours: Alexa Fluor 488 goat anti-rabbit IgG 1:400, Invitrogen #A11008, Alexa Fluor 546 goat anti-mouse IgG 1:400, Invitrogen #A11003, Alexa Fluor 546 goat anti-rat IgG 1:400, Invitrogen #A11081. After 3 times of washing with TBS, sections were quenched in 70% ethanol with 1x TrueBlack Lipofuscin Autofluorescence Quencher (Biotium) for 5 minutes, followed by 2 times washing with TBS and coverslipping with DAPI Fluoromount-G (SouthernBiotech).

A modified immunoperoxidase protocol: a one-in-six series of coronal forebrain sections and a one-in-forty-eight series of coronal spinal cord sections were mounted on 25 x 75 mm microscope slides (Fisher Scientific) and air-dried for 30 minutes. After quenching endogenous peroxidase activity in 1% H_2O_2 and blocking in TBS with 4% Triton X-100 and 15% normal swine or rabbit serum (Vector) for 1 hour, sections were incubated in the following primary antibodies for interneuron markers in % Triton X-100 and 15% normal swine or rabbit serum overnight at 4°C: rabbit anti-PV, 1:1000, SWant #PV27a, rabbit anti-CB 1:1000, SWant #CB38, rabbit anti-calretinin (CR) 1:1000, SWant CR7697, rabbit anti-

Somatostatin (SOM) 1:1000, Immunostar #20067, and rat anti-COUP TF1-interacting protein 2 (CTIP2) 1:1000, abcam ab18465. Subsequently, sections were incubated for 2 hours in biotinylated swine anti-rabbit secondary antibody (1:200, Dako #E0353) or biotinylated rabbit anti-rat secondary antibody (1:200, Vector #BA4001) in TBS with 4% Triton X-100 and 15% normal swine or rabbit serum for 2 hours, followed by 2-hour incubation in Vectastain ABC (avidin-biotin, 1:200, Vector). Immunoreactivity was visualized using 0.05% DAB (Sigma) and 0.005% H_2O_2, followed by airdrying for 2 hours and coverslipping with DPX mounting medium (Electron Microscopy Sciences).

Primers for RT-qPCR

The sequences of primers (5' to 3') used for RT-qPCR are as follows. GFAP, forward ATCGAGATCGCCACCTACAG, reverse CTCACATCACCACGTCCTTG; C3, forward CCAGCTCCCCATTAGCTCTG, reverse GCACTTGCCTCTTTAGGAAGTC; H2-D1, forward TCCGAGATTGTAAAGCGTGAAGA, reverse ACAGGGCAGTGCAGGGATAG; Ligp1, forward GGGGCAATAGCTCATTGGTA, reverse ACCTCGAAGACATCCCCTTT; Srgn, forward GCAAGGTTATCCTGCTCGGA, reverse TGGGAGGGCCGATGTTATTG; Amigo2, forward GAGGCGACCATAATGTCGTT, reverse GCATCCAACAGTCCGATTCT; Serping1, forward ACAGCCCCCTCTGAATTCTT, reverse GGATGCTCTCCAAGTTGCTC; Cd14, forward GGACTGATCTCAGCCCTCTG, reverse GCTTCAGCCCAGTGAAAGAC; Tgm1, forward CTGTTGGTCCCGTCCCAAA, reverse GGACCTTCCATTGTGCCTGG; S100a10, forward CCTCTGGCTGTGGACAAAAT, reverse CTGCTCACAAGAAGCAGTGG; Cd109, forward CACAGTCGGGAGCCCTAAAG, reverse GCAGCGATTTCGATGTCCAC; Ptgs2, forward GCTGTACAAGCAGTGGCAAA, reverse CCCCAAAGATAGCATCTGGA; Clcf1, forward CTTCAATCCTCCTCGACTGG, reverse TACGTCGGAGTTCAGCTGTG.

Quantitative gait analysis

Gait analysis was performed using the *CatWalk XT* (Noldus Information Technology bv, Wagenigen, Netherlands) semi-automated gait analysis system, as described previously (1,2). Briefly, mice were trained at least 2 days prior to data analysis and habituated to the room where behavior was performed overnight before testing. Each mouse needed to have run durations ranging from 1 to 5 seconds to be considered compliant. *Catwalk XT* 10.5 software (Noldus Information Technology) was then used to analyze different parameters associated with gait for each individual paw - Right Fore, Left Fore, Right Hind or Left Hind, in addition to removing any unwanted traces due to other parts of the body (e.g. mouse's belly, scrotum or tail). Data for individual limbs were then averaged across all four paws in each run for individual animals where average data are shown. These data were then exported into an MS Excel spreadsheet (Microsoft, Seattle, WA).

Neurotransmitter analysis

The concentration of cortical neurotransmitters including glutamate (Glu), glutamine (Gln), and GABA was measured by liquid chromatography with tandem mass spectrometry (LC-MS/MS). Mouse cortex samples (n=4, $Cln2^{R207X}$ and WT mice at 3 months of age) were homogenized in water (10 mL/g tissue). All amino acids listed above were extracted from 20µL of homogenate with 200µL of methanol after addition of 20µL of internal standard solution containing Glu-d_3 (500µg/mL), Gln-$^{13}C_5$ (300µg/mL), and GABA-d_6 (300µg/mL). A seven-point standard curve was prepared in duplicate. The analysis of amino acids was performed on a Shimadzu 20AD HPLC system and a SIL-20AC autosampler coupled to 4000Qtrap mass spectrometer (AB Sciex) operated in positive multiple reaction monitoring (MRM) mode. Data processing was conducted with Analyst 1.6.3 (Applied Biosystems).

Calibrators that deviate by more than 15% of nominal concentrations were excluded from construction of calibration curve, except that deviation of 20% was acceptable for the lower limit of quantification (LLOQ), which is the low end of calibration curve.

TPP1 enzyme activity assays

TPP1 enzyme activity in proteins extracted from forebrain homogenates was performed using fluorometric assays, as described previously (3,4). Briefly, upon removal from -80˚C storage, frozen forebrain and spinal cord tissues were homogenized in lysis buffer [50 mM sodium acetate pH 4.0; 0.1% Triton X-100; 1x HALT Protease Inhibitor Cocktail (Thermo Fisher Scientific)]. Samples were centrifuged at 12,000g for 25 min at 4˚C. Total protein concentration in each supernatant was measured using the Pierce BCA Protein Assay (Thermo Fisher Scientific), using a 96-well plate format. 10 µg of total protein was loaded into each well with 100 µl final volume of 100 µM Ala-Ala-Phe AMC (Sigma Aldrich) diluted in substrate buffer (0.1M sodium acetate pH 4.0; 0.1% Triton X-100). Reactions were carried in the dark at 37˚C for 1h and stopped by adding 50 ml of 100 mM sodium chloroacetate and 20 mM sodium acetate. Fluorescence intensity was measured in a Synergy H1 microplate reader (BioTek; Excitation 380 nm/Emission 460 nm) and the results were normalized to the protein content per assay. Each assay was done in triplicates with the average values used for analysis.

References

1. Nelvagal HR, Dearborn JT, Ostergaard JR, Sands MS, Cooper JD. Spinal manifestations of CLN1 disease start during the early postnatal period. *Neuropathol Appl Neurobiol*. 2021;47(2):251-267. doi:10.1111/nan.12658

2. Hamers FPT, Koopmans GC, Joosten EAJ. CatWalk-Assisted Gait Analysis in the

Assessment of Spinal Cord Injury. *https://home-liebertpub-com.beckerproxy.wustl.edu/neu*. 2006;23(3-4):537-548. doi:10.1089/NEU.2006.23.537

3. Vidal-Donet JM, Cárcel-Trullols J, Casanova B, Aguado C, Knecht E. Alterations in ROS Activity and Lysosomal pH Account for Distinct Patterns of Macroautophagy in LINCL and JNCL Fibroblasts. *PLoS One*. 2013;8(2). doi:10.1371/journal.pone.0055526

4. Geraets RD, Langin LM, Cain JT, et al. A tailored mouse model of CLN2 disease: A nonsense mutant for testing personalized therapies. Langmann T, ed. *PLoS One*. 2017;12(5):e0176526. doi:10.1371/journal.pone.0176526

Supplementary figures and figure captions

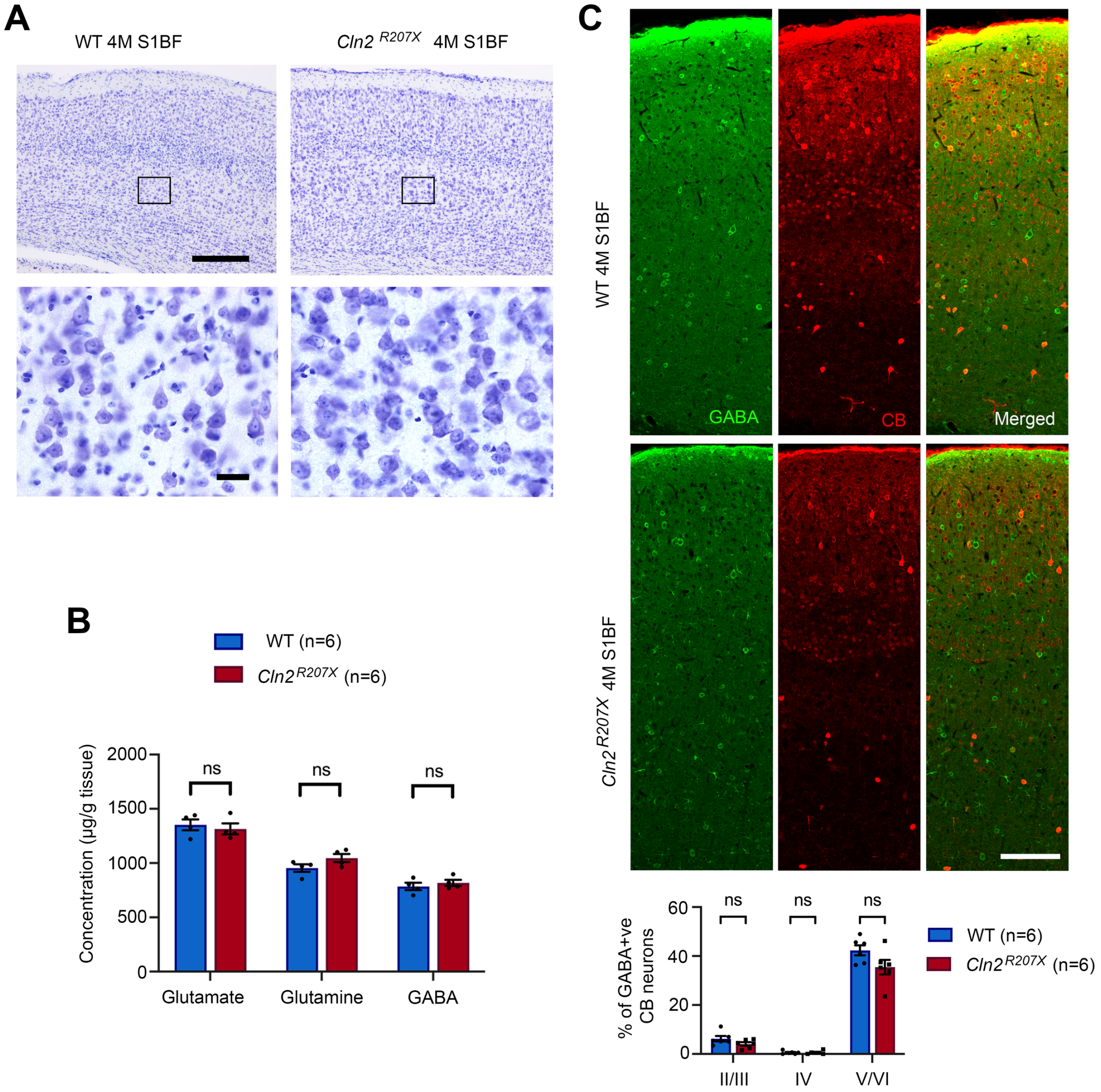

Figure S1. Nissl staining and neurotransmitter analysis in *Cln2*^{R207X} mice. (*A*) Representative examples of Nissl-stained sections through the primary somatosensory cortex (S1BF) of *Cln2*^{R207X} and WT control mice at 4 months. Selected fields (indicated by black boxes) shown at higher magnification the distention of surviving neurons with accumulated storage material is apparent in *Cln2*^{R207X} mice. Scale bars = 200 µm (upper panels) and 20 µm (lower panels). (*B*) Measurement of neurotransmitters including glutamate, glutamine, and GABA in WT and *Cln2*^{R207X} mouse cortex homogenates at 3 months by LC-MS/MS shows no significant difference between genotypes. Values are shown as mean ± SEM (n = 4 mice per group). Dots represent values from individual animals. One-way ANOVA with Bonferroni correction. (*C*) Co-immunostaining reveals the layer-specific expression of GABA (green) in calbindin (CB, red)-positive neurons within S1BF of both WT and *Cln2*^{R207X} mice at 4 months. There was no significant difference in the composition of GABA- and CB- double-positive neurons in layer II/III, IV, and V/IV between genotypes. Scale bars = 200 µm. Values are shown as mean ± SEM (n = 6 mice per group). Dots represent values from individual animals. Multiple t-test with Holm-Šídák correction.

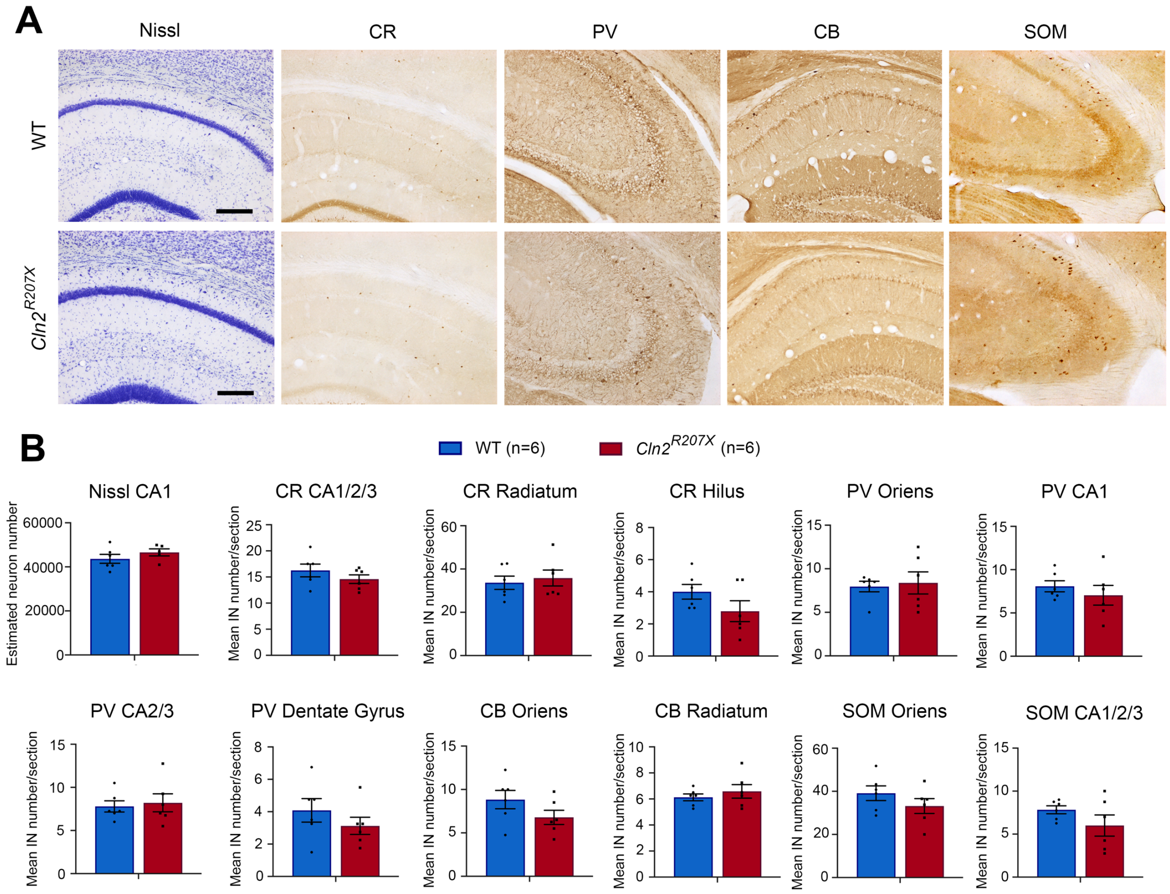

Figure S2. Restricted loss of hippocampal neuron populations in *Cln2*^{R207X} mice. Unbiased stereological counting reveals no change in the number of Nissl-stained neurons within CA1, parvalbumin (PV)-positive interneurons within the stratium oriens, CA1, CA2/3, and dentate gyrus, calretinin (CR)-positive interneurons within CA1/2/3, radiatum, and hilus, calbindin (CB)-positive interneurons within stratum oriens and radiatum, and somatostatin (SOM)-positive interneurons within Oriens and CA1/2/3 of *Cln2*^{R207X} mouse hippocampi compared with age-matched WT controls at 4 months (*A* and *B*). Dots represent values from individual animals. **P* < 0.05, ***P* < 0.01, ****P* < 0.001, *****P* < 0.0001, multiple t-test with Holm-Šídák correction. Values are shown as mean ± SEM (n = 6 mice per group).

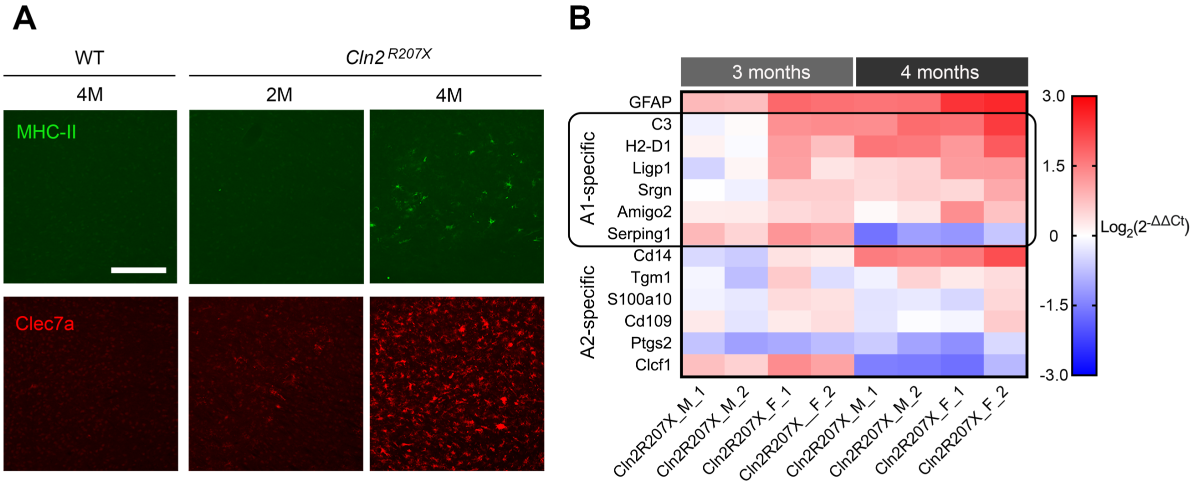

Figure S3. *Cln2*^{R207X} mice show altered expression of multiple markers of microglial activation and astrogliosis. (*A*) Immunostaining for the 'M1' microglial marker MHC class II (MHC-II, green) and the 'M2' or disease-associated microglial (DAM) marker Clec7a (red) reveals increased immunoreactivity for these markers within the ventral posterior nuclei of thalamus (VPM/VPL) of *Cln2*^{R207X} mice at 4 months. Scale bar = 200 µm. (*B*) RT-qPCR analysis shows a limited expression of A1/A2 astrocyte-specific genes in *Cln2*^{R207X} mouse forebrains compared with WT control mice at 3 and 4 months.

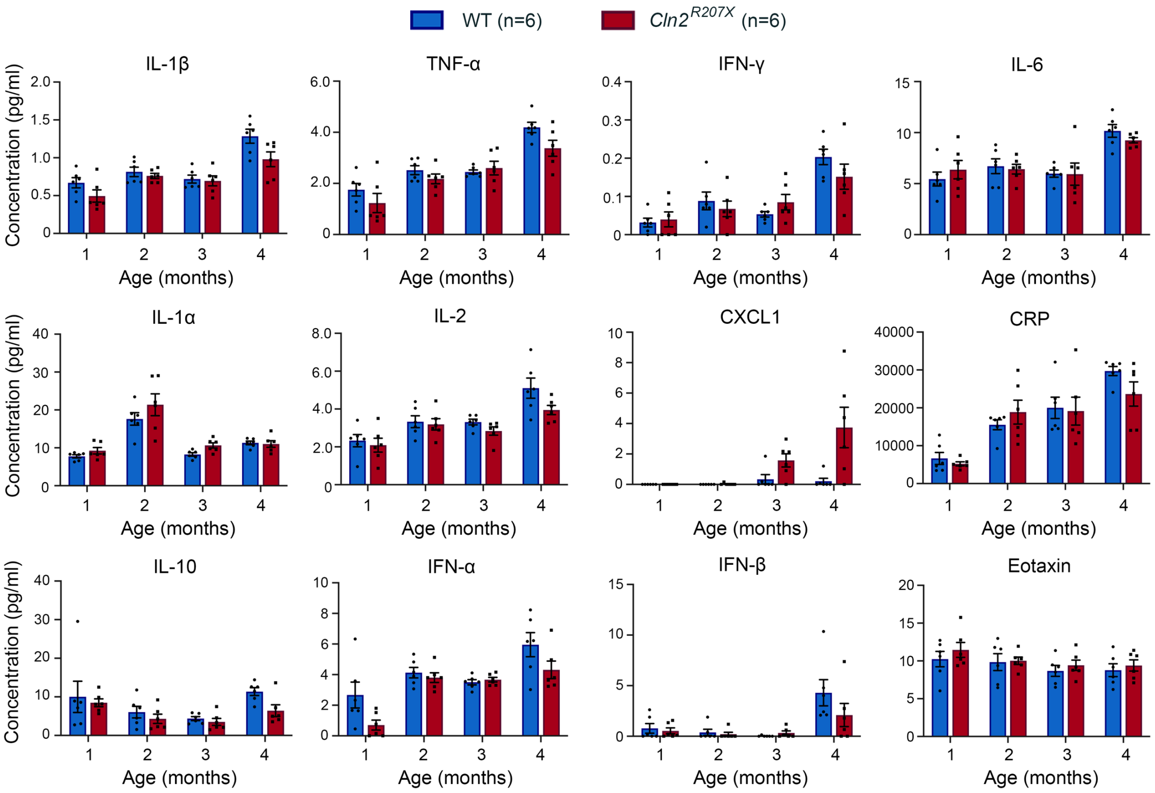

Figure S4. ***Cln2^{R207X}*** **mouse forebrains show distinct neuroinflammatory changes.** Observed concentrations (pg/ml) of multiple cytokines and chemokines measured in forebrain homogenates reveal no significant change in IL-1β, TNF-α, IFN-γ, IL-6, IL-1α, IL-2, CXCL1, CRP, IL-10, IFN-α, IFN-β, and Eotaxin in *Cln2^{R207X}* forebrains (red bars) compared with age-matched WT controls (blue bars). Dots represent values from individual animals. **P* < 0.05, ***P* < 0.01, ****P* < 0.001, *****P* < 0.0001, multiple t-test with Holm-Šídák correction. Values are shown as mean ± SEM (n = 6 mice per group). See Data File S1 for complete data.

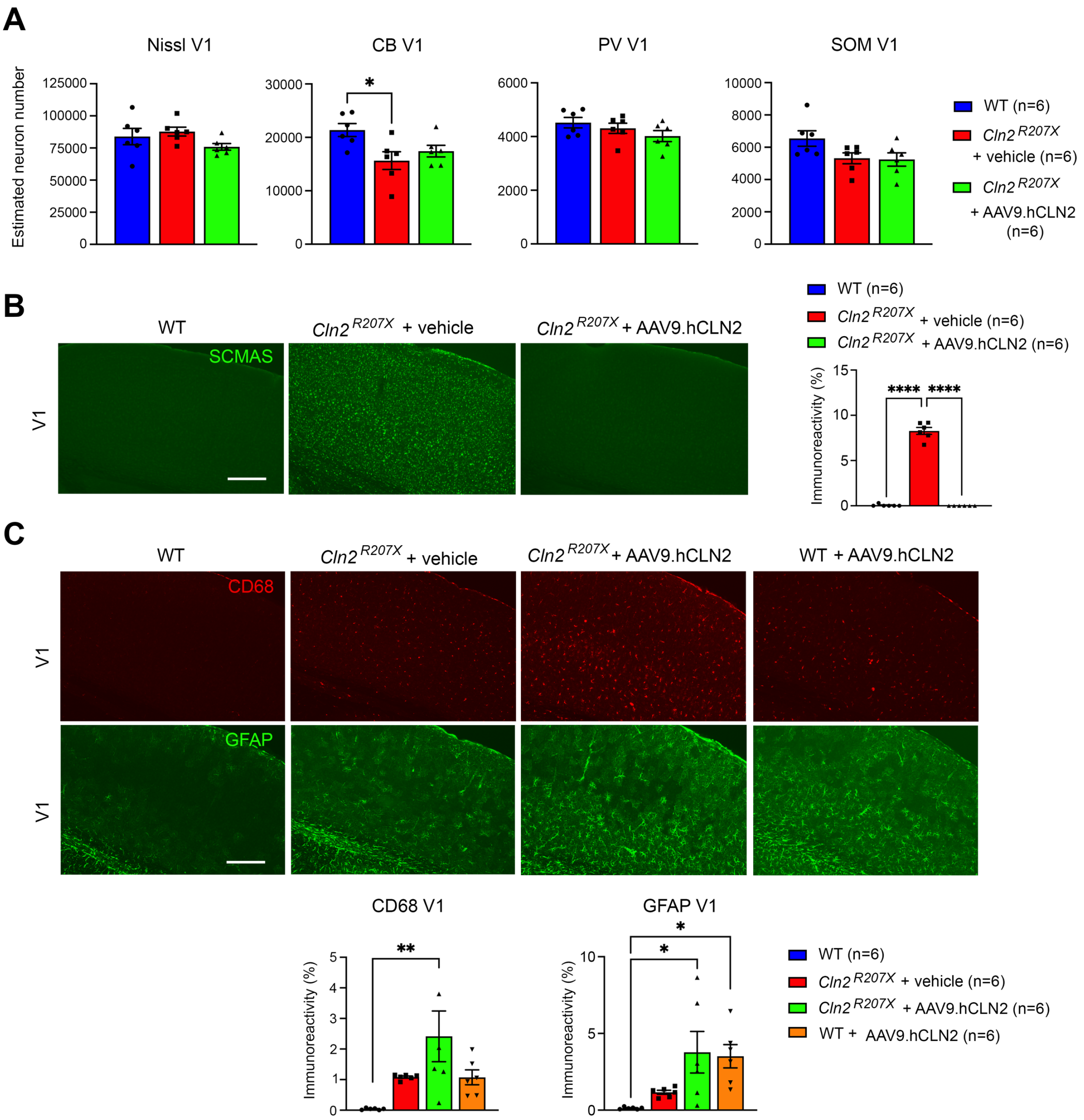

Figure S5. AAV9-mediated gene therapy resulted in an unexpected neuroimmune response in the primary visual cortex. (*A*) Unbiased stereological counts show no significant change in the number of Nissl-stained pyramidal neurons and calbindin (CB)-, parvalbumin (PV)-, and somatostatin (SOM)-positive interneuron populations within the primary visual cortex (V1) of AAV9.hCLN2-treated *Cln2*^{R207X} mice. (*B*) Immunostaining for subunit c of mitochondrial ATP synthase (SCMAS, green), and quantitative thresholding image analysis on immunoreactivity of these markers reveal the complete abrogation of SCMAS accumulation within V1. Scale bar = 200 µm. (*C*) Immunostaining for cluster of differentiation 68 (CD68, red) and glial fibrillary associated protein (GFAP, green) and quantitative thresholding image analysis on immunoreactivity of these markers reveal significantly more microglial activation and astrogliosis within V1 of AAV9.hCLN2-treated *Cln2*^{R207X} mice at 3 months. Scale bar = 200 µm. Dots represent values from individual animals. Values are shown as mean ± SEM (n = 6 mice per group). *$P < 0.05$, **$P < 0.01$, one-way ANOVA with Bonferroni correction.

Cln2^{R207X} mouse ID	Time from last seizure to death
a	29 hours 34 minutes
b	14 seconds
c	2 minutes 42 seconds
d	2 minutes 42 seconds
f	28 minutes 32 seconds
f	1 minutes 3 seconds
g	10 seconds
h	28 seconds
i	22 seconds
j	No seizure

Table 1. Interval between last seizure and death in *Cln2*^{R207X} mice.

Video 1. Video-EEG recordings show a fatal spontaneous seizure in a *Cln2*^{R207X} mouse.

Data File 1. Full sequence of construct including, CB7 promoter, modified sequence of CLN2 cDNA, RBG polyadenylation sequence and the full parental plasmid backbone.

Data File 2. Full results of statistical analyses.
