# Supplementary material for: Gene therapy ameliorates spontaneous seizures associated with cortical neuron loss in a Cln2R207X mouse model
Source: J Clin Invest. 2023 Jun 15;133(12):e165908. doi: 10.1172/JCI165908 (PMC10266778; doi:10.1172/JCI165908)
Supplement: Supplemental data set 1 [file jci-133-165908-s013.pdf]

ctgcgcgctcgctcgctcactgaggccgcccgggcaaagcccgggcgtcgggcgacctttggtcgcccggcctcagtgagcgagcgagcgcgcagag
agggagtggccaactccatcactaggggttccttgtagttaatgattaacccgccatgctacttatctaccagggtaatggggatcctctagaactatag
ctagtcgacattgattattgactagttattaatagtaatcaattacggggtcattagttcatagcccatatatggagttccgcgttacataacttacggta
aatggcccgcctggctgaccgcccaacgacccccgcccattgacgtcaataatgacgtatgttcccatagtaacgccaatagggactttccattgacgt
caatgggtggagtatttacggtaaactgcccacttggcagtacatcaagtgtatcatatgccaagtacgccccctattgacgtcaatgacggtaaatgg
cccgcctggcattatgcccagtacatgaccttatgggactttcctacttggcagtacatctacgtattagtcatcgctattaccatggtcgaggtgagccc
cacgttctgcttcactctccccatctcccccccctccccacccccaattttgtatttatttattttttaattattttgtgcagcgatgggggcgggggggg
ggggggcgcgcgccaggcggggcggggcggggcgaggggcggggcggggcgaggcggagaggtgcggcggcagccaatcagagcggcgcgctc
cgaaagtttccttttatggcgaggcggcggcggcggcggccctataaaaagcgaagcgcgcggcgggcgggagtcgctgcgcgctgccttcgccccg
tgccccgctccgccgccgcctcgcgccgcccgccccggctctgactgaccgcgttactcccacaggtgagcgggcgggacggcccttctcctccgggct
gtaattagcgcttggtttaatgacggcttgtttcttttctgtggctgcgtgaaagccttgaggggctccgggagggccctttgtgcggggggagcggctc
ggggggtgcgtgcgtgtgtgtgtgcgtggggagcgccgcgtgcggctccgcgctgcccggcggctgtgagcgctgcgggcgcggcgcggggctttgtg
cgctccgcagtgtgcgcgaggggagcgcggccgggggcggtgccccgcggtgcggggggggctgcgaggggaacaaaggctgcgtgcggggtgtg
tgcgtgggggggtgagcagggggtgtgggcgcgtcggtcgggctgcaacccccctgcacccccctccccgagttgctgagcacggcccggcttcggg
tgcggggctccgtacggggcgtggcgcggggctcgccgtgccgggcggggggtggcggcaggtgggggtgccgggcggggcggggccgcctcggg
ccggggagggctcgggggaggggcgcggcggcccccggagcgccggcggctgtcgaggcgcggcgagccgcagccattgccttttatggtaatcgt
gcgagagggcgcagggacttcctttgtcccaaatctgtgcggagccgaaatctgggaggcgccgccgcaccccctctagcgggcgcggggcgaagcg
gtgcggcgccggcaggaaggaaatgggcggggagggccttcgtgcgtcgccgcgccgccgtccccttctccctctccagcctcggggctgtccgcggg
gggacggctgccttcgggggggacggggcagggcggggttcggcttctggcgtgtgaccggcggctctagagcctctgctaaccatgttcatgccttct
tcttttcctacagctcctgggcaacgtgctggttattgtgctgtctcatcattttggcaaagaattcacgcgtgccaccatgggactgcaggcctgtctgc
tgggactgttcgccctgatcctgagcggcaagtgcagctacagccccgagcccgaccagagaagaacactgcctccaggctgggtgtccctgggcag
agctgaccctgaagaggaactgagcctgaccttcgccctgcggcagcagaacgtggaaagactgagcgagctggtgcaggccgtgtccgatcctagc
agccctcagtacggcaagtacctgaccctggaaaacgtggccgacctcgtgcggcctagccctctgacactgcacaccgtgcagaagtggctgctggc
tgccggcgctcagaaatgccactccgtgatcacccaggactttctgacctgttggctgagcatccggcaggccgaactgctgctgcctggggccgagtt
tcaccactatgtgggcggacccaccgagacacatgtcgtgcgcagcccacacccttaccagctgccacaggctctggcccctcacgtggactttgtggg
aggcctgcacagattcccccaaccagcagcctgagacagaggcctgagccacaagtgaccggcacagtgggcctgcatctgggcgtgacacctag
cgtgatccggaagcggtacaacctgaccagccaggatgtgggcagcggcaccagcaacaatagccaggcctgcgcccagttcctggaacagtacttc
cacgacagcgatctggcccagttcatgcggctgttcggcggcaacttcgcacatcaggctagcgtggccagagtcgtgggccagcagggaagaggca
gagccggaattgaggcctccctggacgtgcagtacctgatgagcgctggcgccaacatcagcacctgggtgtacagcagccccggcagacacgagg
gccaggaaccttttctgcagtggctgatgctgctgagcaacgagagcgccctgcctcatgtgcacacagtgtcctacggcgacgacgaggacagcctg
agcagcgcctacatccagagagtgaacaccgagctgatgaaggccgctgccaggggactgaccctgctgtttgcctctggcgatagcggagccggct
gttggagtgtgtcaggccggcaccagttcagacccacctttcctgccagctcccccctacgtgacaaccgtgggcggcacctcctttcaggaacccttcct
gatcaccaacgagatcgtggactacatcagcggcggaggcttcagcaacgtgttccccagacccagctaccaggaagaggccgtgaccaagttcctg
tcctccagccctcatctgcccccccagctcctacttcaacgccagcggcagagcctacccagatgtggccgctctgtccgacggctactgggtggtgtcca
acagagtgcccatcccttgggtgtccggcacaagcgccagcacccctgtgtttggcggcatcctgtccctgatcaacgagcacagaatcctgtccggca
gaccccccctgggcttcctgaaccctagactgtaccagcagcacggcgctggcctgttcgatgtgaccagaggctgccacgagagctgcctggacgag
gaagtggaaggccagggcttctgttctggccctggctgggatcctgtgaccggatggggcacccctaacttccccgccctgctgaaaacactgctgaa
cccctgatgactcgaggacggggtgaactacgcctgaggatccgatctttttccctctgccaaaaattatggggacatcatgaagccccttgagcatct
gacttctggctaataaaggaaatttattttcattgcaatagtgtgttggaattttttgtgtctctcactcggaagcaattcgttgatctgaatttcgaccacc
cataatacccattaccctggtagataagtagcatggcgggttaatcattaactacaaggaacccctagtgatggagttggccactccctctctgcgcgc
tcgctcgctcactgaggccgggcgaccaaaggtcgcccgacgcccgggctttgcccgggcggcctcagtgagcgagcgagcgcgcagccttaattaa
cctaattcactggccgtcgttttacaacgtcgtgactgggaaaaccctggcgttacccaacttaatcgccttgcagcacatccccctttcgccagctggc
gtaatagcgaagaggcccgcaccgatcgcccttcccaacagttgcgcagcctgaatggcgaatgggacgcgccctgtagcggcgcattaagcgcggc
gggtgtggtggttacgcgcagcgtgaccgctacacttgccagcgccctagcgcccgctcctttcgctttcttcccttcctttctcgccacgttcgccggctt

tccccgtcaagctctaaatcgggggctccctttagggttccgatttagtgctttacggcacctcgaccccaaaaaacttgattagggtgatggttcacgt
agtgggccatcgccctgatagacggtttttcgccctttgacgttggagtccacgttctttaatagtggactcttgttccaaactggaacaacactcaaccc
tatctcggtctattcttttgatttataagggattttgccgatttcggcctattggttaaaaaatgagctgatttaacaaaaatttaacgcgaattttaacaa
aatcatgtgagcaaaaggccagcaaaaggccaggaaccgtaaaaaggccgcgttgctggcgtttttccataggctccgcccccctgacgagcatcac
aaaaatcgacgctcaagtcagaggtggcgaaacccgacaggactataaagataccaggcgtttccccctggaagctccctcgtgcgctctcctgttcc
gaccctgccgcttaccggatacctgtccgcctttctcccttcgggaagcgtggcgctttctcatagctcacgctgtaggtatctcagttcggtgtaggtcgt
tcgctccaagctgggctgtgtgcacgaaccccccgttcagcccgaccgctgcgccttatccggtaactatcgtcttgagtccaacccggtaagacacga
cttatcgccactggcagcagccactggtaacaggattagcagagcgaggtatgtaggcggtgctacagagttcttgaagtggtggcctaactacggct
acactagaagaacagtatttggtatctgcgctctgctgaagccagttaccttcggaaaaagagttggtagctcttgatccggcaaacaaaccaccgct
ggtagcggtggtttttttgtttgcaagcagcagattacgcgcagaaaaaaaggatctcaagaagatcctttgatcttttctacggggtctgacgctcagt
ggaacgaaaactcacgttaagggattttggtcatgagattatcaaaaaggatcttcacctagatccttttgatcctccggcgttcagcctgtgccacagc
cgacaggatggtgaccaccatttgccccatatcaccgtcggtactgatcccgtcgtcaataaaccgaaccgctacaccctgagcatcaaactcttttatc
agttggatcatgtcggcggtgtcgcggccaagacggtcgagcttcttcaccagaatgacatcaccttcctccaccttcatcctcagcaaatccagccctt
cccgatctgttgaactgccggatgccttgtcggtaaagatgcggttagcttttacccctgcatctttgagcgctgaggtctgcctcgtgaagaaggtgttg
ctgactcataccaggcctgaatcgccccatcatccagccagaaagtgagggagccacggttgatgagagctttgttgtaggtggaccagttggtgattt
tgaacttttgctttgccacggaacggtctgcgttgtcgggaagatgcgtgatctgatccttcaactcagcaaaagttcgatttattcaacaaagccgccg
tcccgtcaagtcagcgtaatgctctgccagtgttacaaccaattaaccaattctgattagaaaaactcatcgagcatcaaatgaaactgcaatttattc
atatcaggattatcaataccatatttttgaaaaagccgtttctgtaatgaaggagaaaactcaccgaggcagttccataggatggcaagatcctggtat
cggtctgcgattccgactcgtccaacatcaatacaacctattaatttcccctcgtcaaaaataaggttatcaagtgagaaatcaccatgagtgacgact
gaatccggtgagaatggcaaaagcttatgcatttctttccagacttgttcaacaggccagccattacgctcgtcatcaaaatcactcgcatcaaccaaa
ccgttattcattcgtgattgcgcctgagcgagacgaaatacgcgatcgctgttaaaaggacaattacaaacaggaatcgaatgcaaccggcgcagga
acactgccagcgcatcaacaatattttcacctgaatcaggatattcttctaatacctggaatgctgttttcccggggatcgcagtggtgagtaaccatgc
atcatcaggagtacggataaaatgcttgatggtcggaagaggcataaattccgtcagccagtttagtctgaccatctcatctgtaacatcattggcaac
gctacctttgccatgtttcagaaacaactctggcgcatcgggcttcccatacaatcgatagattgtcgcacctgattgcccgacattatcgcgagcccat
ttatacccatataaatcagcatccatgttggaatttaatcgcggcctcgagcaagacgtttcccgttgaatatggctcataacaccccttgtattactgtt
tatgtaagcagacagttttattgttcatgatgatatatttttatcttgtgcaatgtaacatcagagattttgagacaccatgttctttcctgcgttatcccct
gattctgtggataaccgtattaccgcctttgagtgagctgataccgctcgccgcagccgaacgaccgagcgcagcgagtcagtgagcgaggaagcgg
aagagcgcccaatacgcaaaccgcctctccccgcgcgttggccgattcattaatgcagctggcacgacaggtttcccgactggaaagcgggcagtga
gcgcaacgcaattaatgtgagttagctcactcattaggcaccccaggctttacactttatgcttccggctcgtatgttgtgtggaattgtgagcggataa
caatttcacacaggaaacagctatgaccatgattacgccagatttaattaaggccttaattagg

ITR, CB7 promoter, CLN2 coding sequence, RBG PolyA Plasmid Backbone
